# Children’s personal values and their behavior in the classroom in the early elementary school years: mapping longitudinal trajectories

**DOI:** 10.1007/s10212-025-00966-2

**Published:** 2025-06-23

**Authors:** Ricarda Scholz-Kuhn, Elena Makarova, Anat Bardi, Lukas F. Litzellachner, Maya Benish-Weisman, Anna K. Döring

**Affiliations:** 1 Institute for Educational Sciences, University of Basel, Hofackerstrasse 30, 4132 Muttenz, Switzerland; 2 Institute for Educational Sciences, University of Basel, Hofackerstrasse 30, 4132 Muttenz, Switzerland; 3 Department of Psychology, Royal Holloway University of London, Bourne and Wolfson Laboratories, Egham, TW20 0EX England; 4 Department of Psychology, University of Bath, Claverton Down, Bath BA2 7 AY, Bath, UK; 5 The Hebrew University of Jerusalem, Mount Scopus, 9190500 Jerusalem, Israel; 6 Centre for Psychological Sciences, University of Westminster, 115 New Cavendish St, London, W1 W 6UW UK

**Keywords:** Personal values, Teacher-rated behavior, Value-behavior relations, Multilevel approach; value development, Longitudinal research

## Abstract

This study pioneers the examination of developmental trajectories of children’s personal values and their behavior in the classroom at the start of elementary school, exploring these dynamics of change in younger children. Additionally, we consider the classroom context, being an important but often overlooked setting for children’s value development. In a sample of 1,184 Swiss children (*Mean*_*age*_ at T1: 6.82 years) who self-reported their values and whose classroom teachers rated their behavior at four time points, set three to four months apart, we analyzed trajectories of personal values and classroom behaviors at both individual and classroom levels. Multilevel growth curve analyses revealed similar trajectories of value change at both individual and classroom levels, showing a substantial linear increase in values of self-transcendence (benevolence and universalism) and a substantial linear decrease in values of self-enhancement (achievement and power). The trajectories of children’s value-expressive behaviors (supportive, achievement-oriented, disciplined, learning-oriented), however, did not align with these value trends and were relatively flat over time, with no significant change at the classroom level. Adding a time-lagged prediction to the multilevel growth curve models, we found that children’s values positively predicted their expressive behavior one time point later, except for conservation values which did not predict future disciplined behavior. Furthermore, the higher children’s supportive, achievement-, learning-oriented, and disciplined behavior was, the higher were their future values of self-transcendence, self-enhancement, openness to change and conservation respectively. Our findings emphasize the bidirectional nature of these relationships, offering important implications for enhancing educational practices in elementary schools.

**Supplementary Information:**

The online version contains supplementary material available at 10.1007/s10212-025-00966-2.

## Introduction

Middle childhood (the age of 6 to 12 years) has been recognized as a crucial period for a child’s psychological, social, and cognitive growth, including the formation of values (Knafo-Noam et al., 2024). However, there are critical gaps in our understanding, particularly regarding the earliest years of primary school (ages 6–8) and the relationship between children’s values and behaviors in the school setting. Only a few studies have focused on longitudinal data to understand developmental trajectories of children’s values (Cieciuch et al., 2016), which can be considered the gold standard in developmental sciences, as it allows researchers to track changes over time (see Daniel et al., 2023). Our study addresses these gaps by focusing on the first two years of primary school in Switzerland and employing a longitudinal design to examine both individual and classroom-level trajectories of values and the behaviors that express them (value-expressive behaviors) across multiple time points. Furthermore, we examine how young children’s values and their behavior predict one another over time to explore the temporal associations between behaviors and values, investigating how each domain predicts the other beyond their respective developmental trajectories.

Through the development of value measures suitable for children, researchers have demonstrated that significant value development takes place during middle childhood (Knafo-Noam et al., 2024). Values are more likely to change frequently in these early years (Twito-Weingarten & Knafo-Noam, 2022), with the rate of change gradually slowing down as individuals approach adulthood (Daniel & Benish-Weisman, 2019). Our study builds on this knowledge by specifically examining the earliest stage of this developmental period. In contrast to the relative stability of values in adulthood (e.g., Leĳen et al., 2022), values are more subject to change throughout childhood as development progresses (e.g., Daniel et al., 2022) and neurological and cognitive advancements such as enhanced abstract thinking (e.g., Harter, 2015) occur, fostering a more intricate understanding of values (Knafo-Noam et al., 2024). Furthermore, at this age, the values of self-transcendence (benevolence and universalism) become more important, while those of self-enhancement (achievement and power) become less important (Daniel et al., 2023), thereby following developmental trajectories in opposite directions.

The school or classroom setting, akin to the family, functions as a microsystem, referring to the immediate environment in which an individual develops (Bronfenbrenner, 2005) and is likely to play an important role in the development of children’s values. The values therein exert a substantial influence on the lives of young people, shaping their self-perceptions, worldview, behavior, and motivation to act in certain ways (Sagiv et al., 2017). Promoting prosocial values among children in school could serve as a strategy to foster a positive school atmosphere, create an optimal learning environment, and addressing issues related to negative and disruptive behaviors in the classroom, a persistent challenge in facilitating effective teaching and learning (Turhan & Akgül, 2017). Although we know that in the elementary school years children’s value priorities are already associated with their behaviors (Abramson et al., 2018; Benish-Weisman et al., 2019; Misgav et al., 2023), only a few studies have explored the relationship between young children’s values and their behaviors in the school setting (e.g., Berson & Oreg, 2016).

### What are values?

**Fig. 1:**
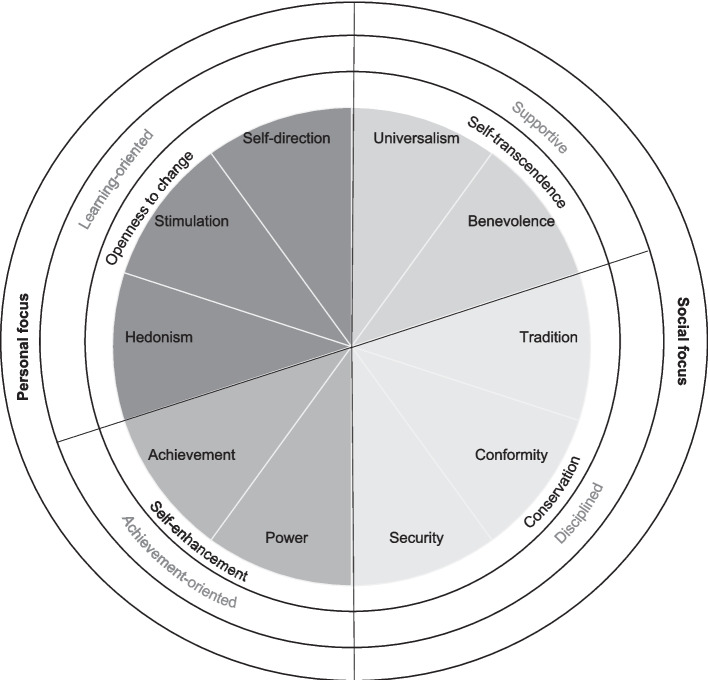
Value-behavior relations (own illustration based on Schwartz’s Values Framework (1992) and prototypical behaviors as formulated by Berson and Oreg (2016))

Values express broad life goals that are important to individuals in life, and they express what individuals strive for (Schwartz, 1992). Values are at the core of a person’s self-concept and identity (Hitlin & Piliavin, 2004), and due to their relation to an individual’s personality traits (e.g., Roccas et al., 2002), attitudes (e.g., Feather, 2004), and behavior (Bardi & Schwartz, 2003), there is variability of values across individuals and also variability in the importance an individual ascribes to different values (i.e., variability within individuals). A common psychological framework in the current scientific literature is Schwartz’s (1992) theory of values. Transferring this well-established theory into the educational field enables us to take a new approach: to study values and their development in the school context. In his values theory, Schwartz (1992) defines the ten value types of universalism, benevolence, tradition, conformity, security, power, achievement, hedonism, stimulation, and self-direction. These so-called basic values are arranged alongside a circular motivational continuum, in which values that are compatible with each other have similar motivational goals and opposing basic values have conflicting motivational goals (see Fig. 1). The ten basic values are subsumed within four broad goals, which are represented by the poles of two bipolar dimensions. The first dimension includes self-transcendence (comprising the basic values of benevolence and universalism) and self-enhancement (comprising power and achievement). The second dimension includes the two poles of conservation (comprising tradition, conformity, and security) and openness to change (comprising hedonism, stimulation, and self-direction). While the higher-order values of self-transcendence and conservation have a social focus, the opposing higher-order values of self-enhancement and openness to change have a personal focus. The following examples were chosen to illustrate the underlying motivations according to the Schwartz’s theory: universalism and benevolence, for instance, share the motivational goal of accepting and helping others (social focus), while achievement and power share the motivational goal of promoting oneself (personal focus). Alongside this continuum, values that are closer to each other are similar, and thus a person who finds universalism important tends to find the neighboring value of benevolence also quite important. Conversely, values that are more distant from one another differ more. For instance, children who prioritize achievement values may focus on outperforming others (personal focus), while giving less consideration to the well-being and welfare of others (benevolence values; social focus). In hundreds of studies, Schwartz’s theory has been confirmed with adult samples (e.g., Sagiv & Schwartz, 2021; Schwartz et al., 2012), adolescence samples (e.g., Benish-Weisman, 2015; Tamm & Tulviste, 2015) and even with children’s samples, where children have been found to have a clear and differentiated understanding of human values (Collins et al., 2017; Döring et al., 2010; Döring & Cieciuch, 2018). However, personal values can differ according to various factors, such as individual characteristics (e.g., gender) (Schwartz & Rubel, 2005), socialization (Leaper & Friedman, 2007), life experience or significant life events (e.g., war, immigration) (Bardi & Goodwin, 2011).

### Values and behavior

Values, as desirable, abstract and trans-situational goals that vary in importance, are understood to motivate behavior (Schwartz, 1994). Many studies have confirmed that values and behavior are systematically related to each other (e.g., see review in Sagiv & Roccas, 2021). For Bardi and Schwartz (2003), people’s expression of value-consistent action is probably a way to naturally pursue their values and to accomplish their goals. This means, for instance, that values relate to behavior in real-life situations (e.g., values predicted voting for political parties (Schwartz, 1996)).

The same motivational conflicts and compatibilities that Schwartz postulated in his values theory (1992) apply among behaviors as well as among value-behavior relations (Twito-Weingarten & Knafo-Noam, 2022) (see Fig. 1). Hence, in the value circle, just like each value is systematically associated to all values, each behavior is systematically associated to all values, and conversely. Multi-dimensional scaling, commonly used to ‘map’ these correlations, indicates that behaviors were positioned close to the values they express share the same motivation with. Behaviors and values with conflicting motivations, i.e., which are not compatible with one another, were located furthest apart (Bardi & Schwartz, 2003).

The term ‘value-expressive behavior’ (Bardi & Schwartz, 2003) refers to actions or choices that individuals make to express and communicate their personal values, beliefs, and identity. The four prototypical value-expressive behaviors ‘supportive’, ‘disciplined’, ‘achievement-oriented’ and ‘learning-oriented’, which were introduced in the literature in a large-scale study of children aged 7 to 11 years at T1 (Berson & Oreg, 2016), correspond to the four higher-order value types of self-transcendence, conservation, self-enhancement and openness to change respectively (see Fig. 1). This implies, for instance, that children who prioritize self-transcendence values over other values are more likely to be supportive, while children who prioritize self-enhancement values are more likely to be achievement-oriented in their ongoing behavior. Given that these relations are consistent with the compatibilities and conflicts in the values’ theory, children who prioritize self-transcendence values are less likely to be achievement-oriented, and children who prioritize self-enhancement values are less likely to be supportive.

Behavior might influence values as well (see Bardi & Goodwin, 2011). In line with this idea, research with adolescents has shown the reciprocal longitudinal relations between values and behavior: Not only did values predict changes in later behavior, but also behaviors predicted later changes in values (Benish-Weisman, 2015). In a study with adolescents in Israel, a longitudinal decline in aggression correlated with a later increase in prioritizing self-transcendence values over time (Benish-Weisman, 2015). Likewise, in a study with Italian adolescents, aggressive behavior predicted a later decline in both self-transcendence and conservation values (Aquilar et al., 2018).

As Bardi and Goodwin (2011) suggested, self-perception theory (Bem, 1967) might explain the link from behavior to values: By observing their behavior, people determine which attitudes and values they find important. This implies that a continuous alteration in behavior can lead to a shift in values. This process of self-observation of one’s own behavior may unfold pathways towards the changing of values. The longitudinal link from behavior to values might be a way to explain value change due to the adjustment to new life situations such as a new behavior due to a new role like becoming a student. Hence, this process might also play a role in the internalization of norms (see Bardi & Goodwin, 2011). However, these associations may be shaped and reinforced by the social environment, including teachers and peers who may interpret children's behavior through the lens of values (see Benish-Weisman, 2019).

Value-behavior links have been demonstrated among adults uncovering that personal values were related to their value-expressive behaviors and negatively related to their opposing value-expressive behaviors (Bardi & Schwartz, 2003). Further, this study found a joint circle structure of values and corresponding value-expressive behaviors located near the values they express, suggesting a systematic pattern of relationships among the two systems. Value-behavior links have also been demonstrated among adolescents, for instance, value-behavior links with anti-social behavior such as aggression (Aquilar et al., 2018; Benish-Weisman, 2015). Evidence for gender differences in value-behavior relations has also been found in adolescents, as seen in studies where girls tend to exhibit more empathetic and supportive intervention strategies in bullying situations, while boys are more inclined towards direct confrontation (e.g., Tamm & Tulviste, 2015).

### Development of children’s values in the elementary school years

The currently sparse research on values in the elementary school years (i.e., approximately the ages of 6- 12 years) shows that important value development occurs during this timeframe (Knafo-Noam et al., 2024). This scarcity of research is partly due to a historic lack of age-appropriate value measures for children, which changed with the development of the Picture-Based Values Survey for Children (PBVS-C; Döring et al., 2010) and later the Animated Values Instrument (AVI; Collins et al., 2017). Through the development and application of these instruments, we now know that five-year-old children already have a meaningful understanding of values that becomes more mature with age (reviewed in Twito-Weingarten & Knafo-Noam, 2022). However, more research is needed, especially given that important neurological and cognitive developments occur during the elementary school years (e.g., greater abstract thinking), and these precursors are likely to be key to the development of a mature understanding of values (reviewed in Knafo-Noam et al., 2024). Indeed, research in 5–12-year-olds found that older children naturally talked about their values in more abstract terms, generalising beyond specific concrete instances (Shachnai & Daniel, 2020). In the early elementary school years (between the ages of 5 and 9 years), children showed growing consistency in their value choices, after which consistency levelled out (Collins et al., 2017). Importantly, during childhood the structure of inter-relations among values gradually became more coherent and in line with the Schwartz (1992) model of human values (reviewed in Twito-Weingarten & Knafo-Noam, 2022). Longitudinal values research showed that at age 10 the individual importance of values is already quite stable, and that stability generally increases further with age (Cieciuch et al., 2016; Daniel et al., 2023; Vecchione et al., 2019).

A review of children’s values (Twito & Knafo-Noam, 2020) also showed increasing similarity with adults over time for some but not all values. Specifically, children increasingly rated self-transcendence as the most important value and self-enhancement as the least important, which aligns with adult perspectives (see Bardi & Goodwin, 2011). However, conservation was ranked higher than openness to change at the beginning of elementary school, while later, openness to change values typically became more important than conservation values in Western cultures. This shift is compatible with an important developmental process during adolescence: establishing autonomy (Branje, 2018). Hence, values change at the beginning of elementary school years, then appear quite stable, but change again towards the end of elementary school. Only a few studies have examined changes in value importance during middle childhood (Daniel et al., 2023), adolescence (Daniel & Benish-Weisman, 2019), and adulthood (Vecchione et al., 2016b). A recent study by Daniel and colleagues (2023) showed that children’s values change in distinct ways as they grow up. Specifically, self-transcendence and self-enhancement values showed the most pronounced trajectories (i.e., patterns of change over time), particularly when the sample was split into two age cohorts. For these values, the models indicated differences between the two cohorts, with the younger children showing more change over time. The importance of self-transcendence values increased in a complex trajectory, while self-enhancement values decreased, both with larger effect sizes in the younger cohort. Openness to change values demonstrated a steady increasing trajectory, while conservation values showed a decreasing trajectory, both with moderate degrees of change. These variations in effect sizes and trajectory patterns across age groups highlight the nuanced development of values during this period. As children get older, their understanding of values becomes clearer, and values that are compatible tend to change in similar ways, while conflicting values change in opposite directions (Daniel et al., 2023). Daniel et al. (2023) focused on individual-level effects, revealing two potential explanations for the observed value trajectories. First, nonlinear developmental patterns suggest more rapid value changes in younger children. Second, the COVID-19 pandemic may have affected the younger cohort more strongly as they experienced it at an earlier developmental stage.

Values are already important guides to behavior in young children (Abramson et al., 2018), but the increasing systematic links between the two have been suggested as a sign of maturity (Knafo-Noam et al., 2024). Indeed, value-behavior links tend to strengthen with age (Abramson et al., 2018a, 2018b; Vecchione et al., 2016a). Following the theoretical suggestion that behavior may affect values (Bardi & Goodwin, 2011), longitudinal directions of predictions between values and behaviors have resulted in a high number of significant longitudinal effects from values to behaviors in the later years of primary school (Vecchione et al., 2016a), providing further evidence for the growing relevance of abstract constructs, such as values, throughout childhood.

Value formation is the product of the interplay of the child’s characteristics (e.g., gender, genes), the wider cultural and societal environment, and contexts close to the child (Twito-Weingarten & Knafo-Noam, 2022). School is an important close context for value formation (e.g., Berson & Oreg, 2016; Hofmann-Towfigh, 2007). Current research argues that, during childhood, understanding of the social world develops, and as values transform from observable to mental, they are possibly better motivators of behavior (Misgav et al., 2023). As children grow up, they are more likely to describe their values using Internal Mental State (IMS) terms, referring to desires, thoughts, and emotions. This maturation in value understanding was observed across all value types, regardless of their focus on social coordination or self-promotion. Interestingly, at Time 2 of the study (*M*_*age*_ T2 = 7.94 years as compared to* M*_*age*_ T1 = 7.25 years), children used more IMS terms to describe unimportant values than important ones. This suggests that children may increasingly conceptualize important values in terms of concrete behaviors, while unimportant values are conceptualized more abstractly. This aligns with the view that individuals conceptualize their values differently in different contexts, with important values being more closely related to everyday behaviors. The study also found a bi-directional association between Theory of Mind (ToM) and IMS use in value descriptions, indicating a co-development of these abilities (Misgav et al., 2023). This developmental perspective suggests that, as children mature, their understanding of values shifts from concrete behaviors to more abstract mental concepts, setting the stage for greater stability in their values and behaviors.

Longitudinal research on values in middle childhood has shown that the values and behaviors of 11-year-old children tend to be relatively stable over time, with values predicting changes in behavior and vice versa (Vecchione et al., 2016a). This stability suggests that, as children's understanding of values becomes more nuanced, the interplay between their values and behaviors also solidifies. Associations of those values and behavior that share the same motivational goals were positive and significant. Other studies on children’s value-behavior relations have focused on specific observed behaviors include sharing behaviors (Abramson et al., 2018), prosocial behaviors (Benish-Weisman et al., 2019), prosocial and aggressive behaviors (Daniel et al., 2020), and helping behavior (Misgav et al., 2023). Nevertheless, there is a notable gap in the literature regarding teacher-rated behaviors for children in the early years of primary school.

Recognizing this gap and the increasing recognition of school as an important social context, our focus now shifts to values in elementary school. In general, the formation of children’s values occurs within multiple social contexts. Bronfenbrenner’s bioecological model offers a systematic conceptualization of these contexts, emphasizing four key components: person, process, context, and time (Bronfenbrenner, 2005; Bronfenbrenner & Morris, 2006). While the family microsystem plays a crucial role in shaping children’s values (e.g., Döring et al., 2017; Makarova et al., 2018), the school context has also proven to significantly influence value development (for macrosystem, e.g., Oeschger et al., 2024a; for mesosystem e.g., Berson & Oreg, 2016; Daniel et al., 2013; Oeschger et al., 2024b; for microsystem e.g., Rubin et al., 2015; Benish-Weisman et al., 2022; Döring et al., 2024).

Analyzing value development within the school context is crucial, as families and schools have complementary roles in socializing children. Adams and Christenson (2000) emphasize that both institutions involve support, teaching, nurturing, punishment, rewards, and evaluation, striving to maintain a delicate balance within the values, beliefs, cultural norms, and societal expectations of their society. Schools play a critical role in reinforcing societal values and acceptable behaviors (Oeschger et al. 2024a), bridging the gap between individual and collective development (e.g., Bronfenbrenner, 1979). Focusing on the classroom level, recent research highlights the importance of considering classroom climate as a key factor in promoting general well-being and positive social-emotional outcomes among young learners. Creating a nurturing and supportive classroom environment is crucial for fostering optimal emotional development in children (Garcia-Peinado, 2023). However, research has traditionally focused more strongly on family as compared to school contexts in children’s value development, leading to a notable gap that our research seeks to address. Eccles and Roeser (2003) highlight that, despite recognizing schools’ importance in cognitive and social development, our understanding of their impact remains limited. Developmental researchers have primarily studied family and peer groups, while educational researchers have focused on schools'influence on intellectual outcomes rather than social-emotional development. This imbalance is particularly evident in value development research, with less attention given to the school context compared to family influences. However, studying the development of values and behaviors in classroom settings is essential due to the significant impact of classroom environments on children’s development. Research has consistently shown that classrooms are key social settings that are important to children’s cognitive, social, and emotional growth (e.g., Hamre & Pianta, 2010; Ornstein et al., 2010; Pianta et al., 2016; Wentzel, 2010). By studying values and behaviors within classroom settings, researchers can gain a comprehensive understanding of how these crucial aspects of child development are shaped by the complex interactions between individual characteristics and the classroom environment. Therefore, the present study focuses on the classroom microsystem, investigating how proximal processes within this context contribute to children’s value development. To understand this impact, recent research provides concrete evidence of the school’s special impact on value expression and development.

Recent research has provided concrete evidence of the school’s unique impact on value expression and development. Berson and Oreg (2016) found that pupils who valued conservation values highly tended to be more disciplined at school, while those who valued openness to change values highly were more learning-oriented, demonstrating a direct link between values and classroom behaviors. Similarly, Benish-Weisman et al. (2022) observed that children’s values have a direct effect on corresponding behaviors in the school setting, except for self-transcendence values. These findings suggest a potential connection between the school environment and children’s value expressions. In the same vein, Daniel et al. (2013) identified six key school-level values across Israeli and European schools, which showed strong correlations with indicators of the school climate for pupils of different ages as well as teachers. Notably, compliance and dominance values were associated with school violence levels, while harmony values were linked to student support measures. These findings highlight the importance of studying school-level value climates and their impact on educational outcomes. The link between values and school climate is mediated by the teachers, who play a crucial role in value transmission through their value-related educational goals, which express the values they would like to see in their pupils (Oeschger et al., 2024b). Recent research by Oeschger et al. (2024a) studied the interplay between teachers'value-related educational goals and school climate. Teachers play a crucial role in value transmission through their value-related educational goals, conveying values via modeling, priming, and discussions (Oeschger et al., 2024b). A longitudinal study in Swiss primary schools found that the school climate of innovation predicted teachers' value-related educational goals of openness to change, with reciprocal effects observed over time (Oeschger et al., 2024a). The overall school climate is equally important, as a positive culture that fosters mutual respect, reduces conflicts, and creates a supportive learning environment (Garcia-Peinado, 2023). This dynamic interplay highlights the need for teachers to reflect on their values to facilitate effective value transmission and social integration of pupils. Additionally, a comparative study between Swiss and UK teachers revealed that value-related educational goals align with national value orientations. UK teachers prioritized conformity and security, while Swiss teachers emphasized self-direction and universalism. The high correlation between teachers'value-related educational goals and national value profiles underscores their role in transmitting societal values (Oeschger et al., 2024b). These findings emphasize the school’s significant effect on children’s value formation and expression, complementing the role of family in this crucial aspect of child development.

### The current study

In this study, we measured children’s values and their teacher-rated behaviors over 1.5 years in early elementary school, with measurement intervals of four months, mirroring the design of comparable longitudinal studies (e.g., Vecchione et al., 2016a). For the first part, we estimated change of values and behavior over time (trajectories), building on existing research of the development of personal values (Daniel et al., 2023), employing the same statistical methods**—**latent growth curve models**—**to investigate how values and behaviors change over time within the classroom environment, focusing on both individual and classroom-level trajectories (**research objective 1**). For the second part, and to understand the dynamic longitudinal relationship between values and behaviors within the school context, we investigated how values predict future behaviors (specifically behaviors at the next time point) and how behaviors predict future values (specifically values at the next time point) in addition to what is explained by their respective developmental trajectories (**research objective 2**). We primarily focused on children’s value development (trajectories of change), for which we derived hypotheses from the literature (Cieciuch et al., 2016; Daniel et al., 2020; Daniel et al., 2023). We also examined the temporal development of classroom behaviors as observed by the teacher (trajectories of change), for which the literature is very scarce and does not yet suggest hypotheses.

Based on these considerations, we propose the following hypotheses for the first part:

*H1a: The importance of self-transcendence and openness to change values increases, whereas the importance of self-enhancement and conservation values decreases in the first two years of primary school.*

*H1b: Individual-level value changes follow Schwartz's value structure (1992), with compatible values changing similarly and conflicting values changing oppositely.*

*H1c: Classroom-level value changes mirror individual-level value changes.*

*H1d: Children’s behavior changes mirror their value changes, with congruent behaviors (those aligned with corresponding values) evolving in similar directions and conflicting behaviors (those in conflict with certain values) changing in opposing directions.*

*H1e: Individual and classroom behavior changes follow the same patterns, with congruent behaviors evolving similarly and divergent behaviors changing oppositely.*

Beyond analyzing the developmental trajectories of values and behavior separately, our study also depicts their dynamic longitudinal relationship within the school context. This study contributes to the literature by examining time-lagged relationships between these variables in the classroom context, applying Berson and Oreg’s (2016) established value-behavior framework. This framework links specific values to corresponding behaviors in schools: conservation with disciplined behavior, self-transcendence with supportive behavior, openness to change with learning-oriented behavior, and self-enhancement with achievement-oriented behaviors (see e.g., Benish-Weisman et al., 2022; Scholz-Kuhn et al., 2023). Research with older children has found bidirectional longitudinal links between values and behavior (i.e., children’s values predict their behavior and vice versa, see e.g., Vecchione et al., 2016a), with a stronger influence from values to behavior (e.g., Benish-Weisman, 2015). However, this relationship may differ in younger children due to their developing ability to conceptualize values as internal mental states. Given previous findings (Misgav et al., 2023), it is reasonable to expect that in younger children, behaviors might be stronger predictors of future values, while in older children, values might become stronger predictors of future behaviors.

Hence, we propose the following hypotheses:

*H2a: Values predict future behavior (specifically behavior at the next time point) in addition to what is explained by their respective developmental trajectories.*

*H2b: Behaviors predict future values (specifically values at the next time point) in addition to what is explained by their respective developmental trajectories.*

## Method

### Participants

The total sample comprised elementary school children from Switzerland (*N*_*overall*_ = 1,342, 49% girls, *M*_*age*_ = 6.82, *SD* = 0.50) who reported their values in four waves of data collection. Data in 2021 and 2022, at four time points, with an interval of three to four months in between. T1 data were collected in March 2021, which falls within the school year that starts in August and ends in the following July. For the present study, data were available for 834 children at T1 (66% of the total sample), 1,184 children at T2 (93%), 1,103 children at T3 (87%) and 1,102 children at T4 (87%), which is an adequate retention rate (see for comparison, Teague et al., 2018). At T1, the sample is smaller because we also tried out a digital version with a third of the children (*N*_*digital*_ = 303), not knowing whether we would be able to conduct the surveys in the classrooms due to the COVID-19-pandemic. We learned through this attempt that the digital tool we used is not appropriate at this age, and therefore the data from these children are not included in the analyses conducted in this article. Overall, children were nested within 96 classrooms, in schools in urban and rural areas of Switzerland. Importantly, all children had the same main classroom teacher across all times of measurement. The average number of children per classroom was 13.4.

### Procedure

Children were recruited through schools. We first sought consent for the project from the authorities of the cantons (formal regions in Switzerland). We then invited schools in these cantons to participate in the research project. Information sheets and consent forms were provided to all parents of children in their first year of elementary school. Only children whose parents provided consent were able to participate in the project. On the day of the data collection, consent was obtained from the children as well. Trained research assistants collected data during two school lessons on the same day. Pupils completed a paper–pencil questionnaire. With the help of a standardized instruction, trained researchers distributed and administered self-report questionnaires in schools, in classroom settings, where all children in the same classroom completed the questionnaires at the same time. If there were any questions or need for clarification, the children could ask the research team at any time. Children received a sticker at each data collection for their participation. While children completed the measures in the classroom, their class teachers completed the behavior questionnaire online. The study was conducted in accordance with requirements of the university’s ethics committee.

### Measures

#### Demographic variables

Students reported their age and gender (coded as 0 = boy, 1 = girl).

#### Children’s values

The Picture-Based Value Survey for Children (PBVS-C, Döring et al., 2010) was used to assess children’s value structure and their priorities. This assessment instrument was designed to meet the cognitive developmental level of younger children (Döring et al., 2010) and has been applied in many studies around the globe (e.g., Abramson et al., 2018; Cieciuch et al., 2016; Döring et al., 2016; Tulviste et al., 2018). Instead of statements like in value measures for adults, pictorial items that visually translate and present concrete behaviors in situations are used to lower the level of abstraction. The PBVS-C comprises two pictures for each of the ten basic values (20 pictures in total), in which a gender-neutral main character carries out a value-relevant action. Children were asked to think of their goals, and they were requested to sort the items (i.e., value stickers) according to their importance in a five-level answer scale. Thus, a ranking of the items takes place, using a forced-choice answer format ranging from 5 “very important” to 1 “not at all important”. Self-transcendence includes universalism and benevolence items, conservation includes tradition, conformity, and security items. Self-enhancement includes power and achievement items, and openness to change includes hedonism, stimulation and self-direction items. The PBVS-C yielded one score on each of the higher-order values per child, which was the average of the items belonging to this higher-order value. Due to its ipsative format, the children’s values are already mean-rate (MRAT) centered by their very nature.

As a preliminary analysis, multidimensional scaling (MDS) was employed to evaluate measuring characteristics (Kruskal & Wish, 1978) and ascertain whether the value structure aligns with Schwartz’s (1992) theoretical model, which suggests a circular organization and the formation of two opposing poles. When children are subjected to abstract components such as pictorial items or Q-sort ranking procedure, as utilized in the Picture-based Value Survey for Children (Döring et al., 2010), the internal consistency tends to be lower (Cieciuch et al., 2016; Döring et al., 2016). Ipsative measures usually produce average negative correlations between items, in contrast to the positive correlations often generated by Likert-type scales, impacting Cronbach’s alpha negatively. As MDS is recommended and used regularly (e.g., Döring et al., 2010), multidimensional scaling was conducted instead of using Cronbach’s alpha. As Borg (2010) suggests, MDS includes various procedures where objects represented as points in a coordinate system in a two-dimensional space are based on Pearson correlations among the importance scores of each pair of values. The distances between points should ideally portray the objects’ proximity. Consequently, a multidimensional scaling analysis was carried out on the matrix of correlations of the 20 items of the PBVS-C (Döring et al., 2010). To evaluate the quality of the MDS solution, we used Stress-1, a normalized measure of the discrepancy between the input proximities and the distances in the MDS configuration. Lower Stress-1 values indicate better fit, with values below 0.20 considered acceptable (Kruskal & Wish, 1978). To affirm the theoretical structure proposed by Schwartz (1992), we conducted separate MDS analyses for the Higher Order Value Types (HOVTs). The results for the first and last point of measurement clearly demonstrated that HOVTs were distinct constructs and that all values were organized in a circular pattern, forming the two opposing poles, and thus confirm the theoretical structure as proposed by Schwartz (1992) (for results see Supplementary Figures S1 and S3). The PBVS-C’s ipsative response format automatically centers value scores, where each child’s average score across the four higher-order value types is zero.

#### Value-expressive behaviors

Children’s value-expressive behaviors in the classroom were rated by teachers, using the 11-item Schoolchildren’s Behavior Scale (Berson & Oreg, 2016). For each participating child, the class teacher completed the whole scale, before moving to the next child in the class. The scale measures children’s supportive, learning-oriented, and achievement-oriented with three items and disciplined behavior with two items. Each of the four behavior indexes express a higher-order value type according to Schwartz (1992), resulting in four categories of value-behavior relations (see Fig. 1). The items, such as “Obeys the rules in class” for disciplined behavior or “Is very competitive in class” for achievement-oriented behavior were rated on a five-point Likert scale from “not at all” to “very much”. Before usage of the scale, we conducted a translation and back translation procedure and also adjusted it to our study’s context (e.g., we replaced “grade” with “assessment”, since first graders in Switzerland do not yet have school grades). The internal consistencies in our study were acceptable (α_T1_= 0.63, α_T2_ = 0.68, α_T3_ = 0.67, α_T4_= 0.71 for supportive behavior; α_T1_= 0.74, α_T2_ = 0.78, α_T3_= 0.75, α_T4_= 0.77 for achievement-oriented behavior; α_T1_ = 0.77, α_T2_ = 0.78, α_T3_= 0.78, α_T4_= 0.79 for learning-oriented behavior; and good α_T1_= 0.80, α_T2_ = 0.85, α_T3_= 0.83, α_T4_= 0.85 for disciplined behavior). Given that the behavior scale (Berson & Oreg, 2016) was modeled over the values scale, multidimensional scaling was further conducted on the behavior scale to affirm the theoretical structure as suggested by Schwartz (1992) (see Fig. 1). The results indicate that behaviors were distinct constructs, being positioned close to the values they express and close with whom they share the same motivation. Behaviors and values with conflicting motivations, i.e., which are not compatible with each other (e.g., self-transcendence values and achievement-oriented behavior), were located most remotely (Bardi & Schwartz, 2003) (for results see Supplementary Figures S2 and S4). To ensure comparability to children’s values and to eliminate individual differences in the use of the response scale, we centered the children’s behaviors, by subtracting the child’s average rating across all behavioral items, following the approach of Bardi and Schwartz (2003). This implies that each child’s average score across all items was zero. This centering was performed prior to including the behavior variables in our analyses.

### Statistical analysis

#### Mapping longitudinal trajectories of values and behavior: Multilevel growth curve models

To address our research objectives, we employed multilevel growth curve models. Addressing *research objective 1*, we examined the developmental trajectories of children’s values and the developmental trajectories of children’s classroom behaviors over time. As in Daniel et al. (2023), we modeled the trajectories through *latent growth curve models*. Importantly, and in addition to the approach used by Daniel et al. (2023), we employed a *multilevel* approach to reflect the hierarchical structure of our data where children are nested within classrooms. This nested structure contained change over time (level 1), nested within individuals (children; level 2), which themselves were nested within groups (classrooms; level 3). Neglecting this nested data structure could result in erroneous conclusions regarding the relationships between variables in the model (e.g., Eid et al., 2017). To recognize the significance of the nested data structure, we calculated intraclass correlation coefficients (ICCs) (see results). Ignoring the hierarchical structure of our sample with ICCs of the observed size would lead to an inflation of the Type-I error (up to 30%, Musca et al., 2011). By employing multilevel modeling, we controlled the less predictable inflation associated with Type-I error through the hierarchical structure, while also managing the more predictable, quantifiable inflation of Type-I error resulting from multiple testing. for the hierarchical structure of the data and *explore both effects at the classroom level and at the individual level* of values and behaviors. Further, we employed growth curve models to estimate the *trajectory* that best reflects the developmental trend in the data, which could be no growth, linear, or quadratic. We estimated which trajectory fit best. Specifically, we conducted two separate analyses: one at the individual level and another at the classroom level. We estimated an overall trajectory across individuals. At the classroom level, we examined how values and behaviors develop across classrooms over time. This analysis focused on identifying classroom-level trajectories of values and behaviors. We aggregated individual-level data to create classroom means for each value and behavior at each time point. These aggregated measures represent the overall level of values and behaviors within each classroom at different points in time. This contextual analysis allowed us to investigate whether classrooms as a whole show distinct developmental patterns. By examining classroom-level trajectories, we could identify trends that might not be apparent when looking at individual-level data alone. For example, we could observe whether certain values or behaviors tend to increase or decrease across classrooms over time, regardless of individual variations within those classrooms. For each of the four higher-order values and their corresponding behaviors, this approach identified the trajectory that best represented the developmental trend across classrooms over the four points of measurement. This multilevel approach enables us to disentangle individual-level changes from classroom-level effects, providing a more nuanced understanding of how values and behaviors develop in the school context.

Addressing our *research objective 2*, we added a time-lagged prediction to the multilevel growth curve models. This allowed us to investigate how children’s values predicted their behavior at the next time point and how children’s behavior predicted their values at the next time point in addition to the prediction through the developmental trajectory. Such time-lagged prediction has been applied in growth curve models in a range of studies in psychology, including pediatric, clinical, social, and personality research (see Hanel et al., 2024; Aafjes-van Doorn et al., 2017; Burns et al., 2015; Duckworth et al., 2010; Grégoire et al., 2021; Madjar et al., 2021; Schlauch et al., 2013; Stephenson et al., 2016). Our syntax is adapted from a publication on value change (Hanel et al., 2024). This strategy allowed us to reveal the overall effect of one predictor (i.e., values respectively behavior) on an outcome variable (i.e., behavior respectively values) at subsequent time point, while controlling for prior levels of the outcome variable. Ultimately, this approach aligned with our study’s aim of understanding how one domain (e.g., behaviors) contributed to the trajectories in another domain (e.g., values) over time. Moreover, we were able to gain insights into the temporal dynamics between predictors and outcomes in the classroom context. This comprehensive modeling approach, combining multilevel structure, growth curve analysis and time-lagged outcomes, provided us with a sophisticated tool to address our research objectives. It allowed us to capture the complex, nested nature of our data, model developmental trajectories, account for individual and group-level variations, and investigate temporal predictive relationships between variables. To disaggregate the total effect into the within-classroom component and the between-classroom component (Curran & Bauer, 2011), we applied Cluster Mean Centering (CMC) where we centered each child’s values and each child’s behaviors around the mean in their classroom.

We employed the R-package nlme (Pinheiro et al., 2021) to fit our models. Initially, we compared no growth, linear, and quadratic growth models to identify the optimal functional form of the trajectories over time from T1 to T4. The no growth model included only a random intercept per individual, respectively classroom, without random slopes over time. The linear model featured linear random slopes over time. The quadratic curve model extended the linear model by including an additional random coefficient β^2^ (the square of time), allowing trajectories to follow a quadratic curve. In the quadratic growth curve model, both time and time-squared were included as predictors to capture nonlinear patterns of change over time. These eight models (4 × for value trajectories, 4 × for behavior trajectories) addressed our first research objective, where we estimated one trajectory across individuals as well as one trajectory across classrooms.

To address our second research objective, we again identified the optimal growth model, this time incorporating time-lagged outcomes and specifically examining the trajectories from T2 to T4 for these outcomes. Having established the optimal growth model specifications, we then added predictors from T1 to T3. First, we predicted behaviors from T2 to T4 based on values from T1 to T3, assessing how values from previous time points predicted future behaviors. Second, we predicted values from T2 to T4 based on behaviors from T1 to T3, assessing how behaviors from previous time points predicted future values. In every model, values were combined with their respective behavior: self-transcendence values with supportive behavior, self-enhancement values with achievement-oriented behavior, conservation values with disciplined behavior, and openness to change values with learning-oriented behavior. We calculated an overall effect across the three prediction intervals (T1 to T2, T2 to T3, and T3 to T4). This analysis provided one coefficient across all times, for all the predictions from earlier time one variable to later time of the other variable. This resulted in single scores that capture how behaviors contribute to the trajectory of values and vice versa, beyond their own developmental patterns. In other words, we aimed to assess how behaviors from previous time points contribute to the trajectory of values at later time points, above and beyond what could be explained by the values’ own trajectories, and conversely, how values from previous time points contribute to the trajectories of behaviors at later time points, beyond what could be explained by the behaviors’ own trajectories. We conducted eight multilevel growth curve models with time as both fixed and random effects, which are important for controlling past effects on the outcome variable.

We centered time at 0, aligning the growth curve intercept with each pupil’s baseline values or behaviors at the first data collection point. This allows for a clearer interpretation of growth trajectories, as the intercept of the growth curve now represented the initial status of each pupil at the beginning of the study. In our models, the slopes present linear and quadratic growth. The linear growth, representing the fixed effect of time, is the average magnitude of change in the outcome variable across the entire sample. This effect reflects the systematic and consistent change in the dependent variable over time, which is the common trend across all individuals in the study, i.e., if children’s behaviors or values increase or decrease over time. In other words, it shows the general trend of the stability of the outcome variable. We used significance tests to compare all calculated models (Eid et al., 2017). To account for missing values and avoid a loss of statistical power, we applied the maximum likelihood algorithm. This approach allows us to utilize all available data points, providing robust parameter estimates while effectively managing the missing data (Curran et al., 2010; see Tables 2–3). A commented R code for the main analysis can be found in the Supplementary Materials (Code S1).

## Results

### Descriptive statistics and correlational analyses

Table 1 presents the means and standard deviations. Correlations for the variables investigated across the study period are presented in Supplementary Tables S1 and S2. The pattern of means suggests that the most important higher-order value was self-transcendence, and the least important value was self-enhancement, which is the same as usually found in adults (Schwartz & Bardi, 2001). While the importance of self-transcendence, conservation and openness to change values, as well as all behaviors, was either stable or increased over time, self-enhancement values showed a decrease with time. The pattern of correlations for basic personal values as well as value-expressive behaviors was in line with Schwartz’s value theory, indicating that higher-order values or behavior types that are opposite in the circle showed negative and significant within-time correlations (e.g., self-enhancement versus self-transcendence).

**Table 1:** Means and standard deviations of variables

		T1	T2	T3	T4
	Variable	*M* *(SD)*	*M* *(SD)*	*M* *(SD)*	*M* *(SD)*
Values	Self-enhancement	−0.38 (0.68)	−0.61 (0.64)	−0.75 (0.60)	−0.79 (0.57)
	Self-transcendence	0.42 (0.52)	0.52 (0.50)	0.59 (0.49)	0.58 (0.49)
	Conservation	−0.01 (0.40)	0.04 (0.39)	0.06 (0.39)	0.04 (0.41)
	Openness to change	−0.03 (0.40)	0.02 (0.42)	0.05 (0.42)	0.09 (0.43)
Behavior	Achievement	−0.13 (0.60)	−0.12 (0.60)	−0.10 (0.58)	−0.04 (0.62)
	Supportive	0.19 (0.66)	0.18 (0.67)	0.21 (0.63)	0.12 (0.58)
	Disciplined	0.19 (0.66)	0.18 (0.67)	0.21 (0.63)	0.12 (0.58)
	Learning	0.15 (0.44)	0.11 (0.46)	0.09 (0.44)	0.08 (0.47)

### Intraclass correlation coefficients (ICCs)

**Fig. 2:**
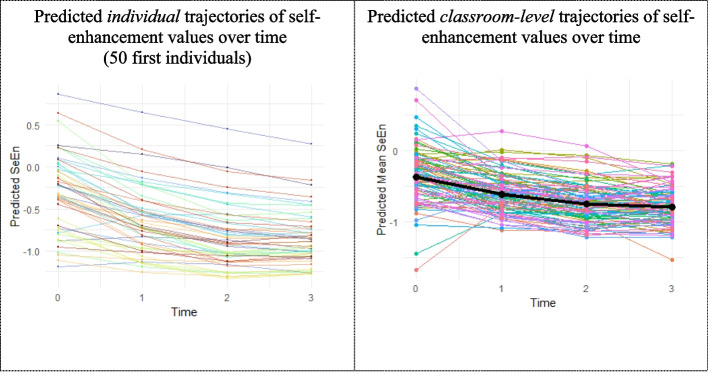
Individual and classroom-level trajectories of self-enhancement values: Visualizing nested developmental patterns

In multilevel models, the intraclass correlation coefficients (ICCs) quantify the proportion of total variance attributable to group-level differences, with higher values indicating greater variability between classrooms in relation to variability within classrooms (Snijders & Bosker, 2012). Our analyses revealed substantial ICCs ranging for the values from 0.07 for conservation and openness, 0.08 for self-transcendence to 0.11 for self-enhancement, and for the behaviors from 0.06 for learning-oriented, 0.09 for disciplined to 0.11 for achievement-oriented and supportive, providing strong justification for using multilevel modeling. Ignoring this clustering could lead to significant Type I error rate inflation, potentially up to 30% in some simulations (up to 30% in simulations, Musca et al., 2011). These ICCs indicate considerable differences between classes that must be accounted for in our statistical approach. To illustrate the complex dynamics of children’s value and behavior development in educational settings, we present two graphs depicting both individual-level and classroom-level trajectories of change (see Fig. 2). These visualizations aid in understanding the nested structure of our data, as evidenced by the high ICCs observed. In our graphs, differences in intercepts between individual and classroom-level trajectories reflect variations in initial levels, while slope differences represent divergent rates of change over time (e.g., Lushin et al., 2020). These visual disparities correspond to the statistical concept of ICC, providing an intuitive representation of the variance explained by class membership (Musca et al., 2011). For instance, Fig. 2 showcases the trajectories for self-enhancement values, which exhibited the highest ICC of 0.11. Here, the distinct patterns in classroom-level trajectories compared to individual trajectories underscore the substantial effect of classroom factors on student outcomes (i.e., values), reinforcing the importance of accounting for the hierarchical nature of educational data in our analyses (Devine et al., 2024).

### Multilevel growth curve models

#### Individual and classroom-level trajectories of change in children’s values and behaviors

In the first step, we analyzed children’s individual and classroom-level trajectories of change in values over time. The quadratic model explained additional variance over the no growth and the linear growth model for all four tested value predictions, for both individual and classroom-level trajectories (*p* < 0.001) (see Table 2). However, for the model with openness to change values, the quadratic model showed non-significant effects, while the linear model showed significant effects. Thus, since the quadratic model does not provide substantial improvement in explaining the data and lacks significant effects, we maintained the simpler linear model for openness to change values, while modeling quadratic growth models for the other three models.

**Table 2:** Results of latent growth models (individual and classroom-level) value trajectories (only best fitting models are presented)

	Self-enhancement		Self-transcendence		Openness to Change		Conservation	
Parameter	Individual	Classroom	Individual	Classroom	Individual	Classroom	Individual	Classroom
**Fixed effects**	Estimate (*SE*)	Estimate (*SE*)	Estimate (*SE*)	Estimate (*SE*)	Estimate (*SE*)	Estimate (*SE*)	Estimate (*SE*)	Estimate (*SE*)
Intercept	−0.36*** (0.03)	−0.35*** (0.04)	0.42*** (0.02)	0.42*** (0.03)	−0.02 (0.01)	−0.02 (0.02)	−0.01 (0.02)	−0.01 (0.02)
Linear slope	−0.28*** (0.03)	−0.29*** (0.04)	0.12*** (0.02)	0.13*** (0.03)	0.04*** (0.01)	0.04*** (0.01)	0.07*** (0.02)	0.07** (0.03)
Quadratic slope	0.05*** (0.01)	0.05*** (0.01)	−0.02*** (0.01)	−0.03** (0.01)			−0.02** (0.01)	−0.02** (0.01)
**Random effects**	Variance	Variance	Variance	Variance	Variance	Variance	Variance	Variance
Level 2 (Individuum)								
Intercept	0.31		0.15		0.08		0.08	
Linear slope	0.19		0.08		0.00		0.08	
Quadratic slope	0.01		0.01				0.01	
Level 3 (Classroom)								
Intercept	0.03	0.14	0.01	0.08	0.00	0.02	0.01	0.06
Linear slope	0.02	0.15	0.01	0.10	0.00	0.01	0.01	0.07
Quadratic slope	0.00	0.01	0.00	0.01			0.00	0.00
Residual	0.13	0.00	0.10	0.00	0.08	0.01	0.07	0.00

*Note*. N = 4223 observations, nested within 1282 individuals and 96 classes. *SE* = Standard Error. *p < 0.05; **p < 0.01; ***p < 0.001

As hypothesized (H1a) self-enhancement values showed a declining trend, starting below average and decreasing at a decelerating rate. Conversely, self-transcendence values began above average and increased gradually with a slight deceleration. Openness to change values demonstrated a steady, modest increase from an average initial point. Conservation values similarly exhibited a gradual increase that slowed over time. Furthermore, we found support for H1b, revealing distinct trajectories for different value types across time, aligning with Schwartz’s value structure (1992), with compatible values changing similarly and conflicting values changing oppositely, and in line with previous research (e.g., Cieciuch et al., 2016).

**Fig. 3:**
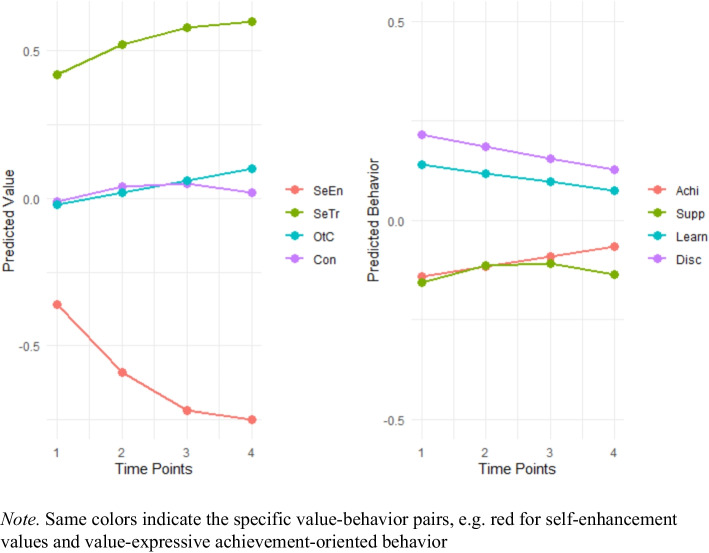
Visualization of individual trajectories of change of children’s values (left) and classroom behaviors (right) over time

Notably, individual and classroom-level models exhibited consistent patterns, supporting H1c. Both levels showed a significant linear decrease in self-enhancement values and a significant linear increase in self-transcendence values over time. Furthermore, both levels showed a significant linear increase in openness to change, while the trajectory for conservation values was characterized by a significant positive linear slope and a significant negative quadratic slope, indicating an initial increase that decelerated over time, resulting in a curvilinear pattern (see Table 2 and Fig. 3).

In the next step, we analyzed children’s individual and classroom-level trajectories of change in *classroom behavior* over time (see Table 3 or Fig. 3). The quadratic model explained additional variance over the no growth and the linear growth model for all four tested behavior predictions considering individual trajectories of change (*p* < 0.001). However, for three models, the slopes showed non-significant effects for quadratic growth, while the linear growth showed a statistically significant effect. Thus, and as above, we maintained the simpler linear model for achievement-oriented, disciplined, and learning-oriented behavior, while modeling quadratic growth models for supportive behavior (see Table 3).

**Table 3:** Results of latent growth models (individual and classroom-level) behavior trajectories (only best fitting models are presented)

	Achievement		Supportive		Learning		Disciplined	
Parameter	Individual	Classroom	Individual	Classroom	Individual	Classroom	Individual	Classroom
**Fixed effects**	Estimate (*SE*)	Estimate (*SE*)	Estimate (*SE*)	Estimate (*SE*)	Estimate (*SE*)	Estimate (*SE*)	Estimate (*SE*)	Estimate (*SE*)
Intercept	−0.14*** (0.02)	−0.10*** (0.02)	−0.16*** (0.02)	−0.13*** (0.02)	0.14*** (0.01)	0.12*** (0.02)	0.21*** (0.03)	0.15*** (0.02)
Linear slope	0.02** (0.01)		0.06* (0.03)		−0.02*** (0.01)		−0.03*** (0.01)	
Quadratic slope			−0.02* (0.01)					
**Random effects**	Variance	Variance	Variance	Variance	Variance	Variance	Variance	Variance
Level 2 (Individuum)								
Intercept	0.21		0.22		0.11		0.28	
Linear slope	0.01		0.01		0.00		0.00	
Quadratic slope			0.00					
Level 3 (Classroom)								
Intercept	0.03	0.05	0.02	0.14	0.01	0.02	0.04	0.06
Linear slope	0.00		0.04	0.22	0.00		0.00	
Quadratic slope			0.00	0.02				
Residual	0.12	0.03	0.13	0.00	0.08	0.01	0.14	0.03

*Note*. Individual Level: *N* = 4032 observations, nested within 1283 individuals and 96 classes. *SE* = Standard Error. *p 0.05; **p 0.01; ***p 0.001

We found a quadratic increase in supportive behavior, suggesting that this behavior was increasing over time (positive linear growth estimate), but since the quadratic growth estimate was negative, it also indicated that the rate of growth initially accelerated but then slowed down over time. Meanwhile, the analyses revealed a linear increase in achievement-oriented behavior, indicating that this behavior showed a steady and constant increase over time. For both disciplined behavior and learning-oriented behavior, the analyses revealed linear decrease, indicating that these behaviors showed a steady and constant decrease over time, where the rate of decrease remained the same. Contrary to our expectations, our analysis showed that children’s behaviors change did not follow the same structure as children’s values, where congruent behaviors evolve in a similar direction, while behaviors that express conflicting values change in opposing directions, and hence we did not find support for our hypothesis H1d.

Next, we analyzed classroom-level trajectories of change in children's behaviors. In all four models, the no-growth models demonstrated the best fit, indicating that the trajectories of supportive, achievement-oriented, learning-oriented, and disciplined behaviors remained constant throughout the study. Similar to the individual level analysis, and contrary to our expectations, we found no support for our hypothesis H1e. This suggests that individual changes within the classes may balance each other out on average, resulting in constant behavior at the class level throughout the study. In sum, the trajectories of children’s value-expressive behaviors did not align with these value trends and were relatively flat over time, with no significant change at the classroom level.

Our contextual analysis, which examined trajectories at the classroom level in addition to the individual level, revealed important distinctions in the developmental patterns of values and behaviors. For values, we observed consistent patterns across both levels, with nearly identical estimates for individual and classroom trajectories. This consistency suggests that value development follows robust patterns that are similar whether examined at the individual or classroom level. However, for behaviors, we found notable differences between individual and classroom-level trajectories. 

While individual-level analyses revealed modest yet statistically significant changes over time – including a subtle quadratic increase in supportive behavior, minor linear increases in achievement-oriented behavior, and slight linear decreases in disciplined and learning-oriented behaviors. These trajectories were considerably weaker than those observed in value-related measures, especially trajectories in self-enhancement and self-transcendence which showed the biggest effects. Crucially, classroom-level behavioral patterns remained stable throughout the study period, with no meaningful fluctuations detected. Given the limited magnitude and inconsistency of these changes, we advise against drawing strong conclusions about systematic behavioral shifts based on these subtle patterns. This discrepancy highlights the importance of our multilevel approach, as it reveals that individual-level changes in behavior may balance out when aggregated at the classroom level. These findings underscore the complex nature of value and behavior development in educational settings. While values seem to develop similarly across individuals and classrooms, behaviors show more variability, with individual changes not necessarily translating to classroom-level trends.

The random effects analysis showed higher individual-level variability in intercepts compared to classroom levels across all behaviors, particularly for disciplined behaviors (see Table 3). Slope variances were minimal, and classroom-level residual variances were low, indicating that individual differences in baseline behaviors dominate over classroom influences. This suggests that developmental trajectories in these behaviors are primarily shaped by individual factors rather than classroom dynamics.

#### Time-lagged associations between children’s values and teacher-rated classroom behaviors

In four models, the time-varying predictors were the values corresponding to the outcome variable (e.g., self-transcendence values and supportive behavior). Outcome variables were time-lagged relative to predictors, meaning that values measured at time *n* were related to classroom behavior at time *n* + 1 to establish temporal precedence. Specifically, we first estimated the outcome trajectory from T2 to T4 and then added the predictor data from T1 to T3. To test H2b, we also examined the opposite temporal sequence. Namely, classroom behaviors at time *n* predicting time-lagged values (measured at time *n* + 1).

In partial support with H2a, we found that children’s values significantly positively predicted the respective behavior of achievement-oriented, supportive, and learning-oriented one time point later, above and beyond what could be explained by the behaviors’ own trajectories. However, conservation values did not significantly predict future disciplined behavior. (see Table 4). This implies that in three out of four value-behavior sets, we found that values from previous time points (T1-T3) positively predicted future behaviors (T2-T4) one time point later above and beyond what could be explained by the behaviors’ own trajectories. This indicates that we found that children’s self-transcendence values at previous time points positively predicted future supportive behavior one time point later, beyond what could be explained by the supportive behavior’s own developmental pattern.

**Table 4:** Time-lagged relationships: Prediction of future behaviors by values (only best fitting models are presented)

	Future Behavior											
	Achievement			Supportive			Learning			Disciplined		
**Fixed effects**	*b*	*SE*	*p*	*b*	*SE*	*p*	*b*	*SE*	*p*	*b*	*SE*	*p*
Intercept	−0.17	0.04	< 0.001	−0.15	0.03	< 0.001	0.09	0.01	< 0.001	0.26	0.04	< 0.001
Linear slope	0.06	0.01	< 0.001							−0.05	0.01	< 0.001
Quadratic slope												
Values	**0.08**	0.02	< 0.001	**0.06**	0.02	0.008	**0.04**	0.02	0.038	0.05	0.03	0.076
**Random effects**	Variance			Variance			Variance			Variance		
Level 2 (Individuum)												
Intercept	0.22			0.20			0.12			0.32		
Linear slope	0.01									0.00		
Quadratic slope												
Level 3 (Classroom)												
Intercept	0.06			0.04			0.01			0.09		
Linear slope	0.01									0.01		
Quadratic slope												
Residual	0.11			0.13			0.08			0.12		

*Note*. In every model, behaviors were combined with their respective values: achievement-oriented behavior with selfenhancement values, supportive behavior with self-transcendence values, learning-oriented behavior with openness to change values, and disciplined behavior with conservation values. Significant effects are bold. Number of observations: 2698, Number of groups: 96 classes, 1170 individuals

In line with H2b, we found that children’s behaviors significantly positively predicted the respective values of self-transcendence, self-enhancement, openness to change, and conservation respectively one time point later above and beyond what could be explained by the values’ own trajectories. This implies that the higher children’s supportive, achievement, learning-oriented and disciplined behavior was, the higher were their future values of self-transcendence, self-enhancement, openness to change and conservation respectively (see Table 5). This indicates that in all four value-behavior sets, children’s behaviors significantly predicted values in subsequent measurements, above and beyond what could be explained by the value’s own trajectories. We calculated an overall effect across multiple prediction intervals (T1 to T2, T2 to T3, and T3 to T4). Taking the same example as above, children’s supportive behavior at previous time points positively predicted future self-transcendence values at later time points above and beyond what could be explained by the self-transcendence values’ own developmental patterns.

**Table 5:** Time-lagged relationships: Prediction of future values by behavior (only best fitting models are presented)

	Future Values											
	Self-Enhancement			Self-Transcendence			Openness to Change			Conservation		
**Fixed effects**	*b*	*SE*	*p*	*b*	*SE*	*p*	*b*	*SE*	*p*	*b*	*SE*	*p*
Intercept	−0.53	0.04	< 0.001	0.50	0.03	< 0.001	−0.04	0.02	0.059	0.04	0.01	0.007
Linear slope	−0.12	0.02	< 0.001	0.03	0.01	0.003	0.04	0.01	< 0.001			
Quadratic slope	0.02	0.01	0.014									
Behavior	**0.09**	0.02	< 0.001	**0.05**	0.02	0.003	**0.08**	0.02	< 0.001	**0.05**	0.01	< 0.001
**Random effects**	Variance			Variance			Variance			Variance		
Level 2 (Individuum)												
Intercept	0.28			0.14			0.09			0.07		
Linear slope	0.02			0.01			0.00					
Quadratic slope	0.00											
Level 3 (Classroom)										0.01		
Intercept	0.08			0.03			0.01					
Linear slope	0.00			0.01			0.00					
Quadratic slope	0.00											
Residual	0.13			0.10			0.07			0.07		

*Note*. In every model, values were combined with their respective behavior: self-enhancement values with achievement-oriented behavior, self-transcendence values with supportive behavior, openness to change values with learning-oriented behavior, and conservation values with disciplined behavior. Significant effects are bold. Number of observations: 2698, Number of groups: 96 classes, 1170 individuals

## Discussion

This research provides novel and important insights into the bidirectional longitudinal predictions of values and behaviors of young children right at the beginning of school. As our design followed children throughout their initial two years in elementary school, we were able to analyze an important developmental period in which social and cognitive changes occur, taking a psychological perspective. From an educational perspective, this study builds on existing research regarding value development in middle childhood (Daniel et al., 2023) by extending the investigation of the longitudinal relationships between values and their expressive behaviors to the Swiss context and younger ages. The study uniquely examines both individual-level changes in children and classroom-level dynamics using a multilevel modeling approach. While primarily focused on children’s value development, our study contributes novel insights by examining the temporal development of classroom behaviors, a crucial factor in school settings. This dual-level analysis offers insights into the potential interactions between classroom environments and individual pupil changes, contributing to the broader discourse on educational effectiveness and child development. Our results converge with previous research that children’s values were associated with their behavior, both being organized in a circular motivational continuum, forming the two opposing poles as suggested by Schwartz (1992, 1994) and this can be found in the school context (i.e., Abramson et al., 2018; Benish-Weisman et al., 2022; Berson & Oreg, 2016). Assessing young children’s values may be more challenging to measure, because they first must be consolidated in children at this young age. However, the replication of the value and behavior structure in this sample shows that the children have understood the picture-based value questionnaire and that they have a reasonable understanding of values.

### Individual and classroom-level trajectories of change in children’s values and behaviors

Our first objective was to identify individual and classroom-level trajectories of change of children’s personal values and their classroom behaviors within the school context. Adding to previous research (e.g., Cieciuch et al., 2016; Daniel et al., 2023) and in line with our expectations, children’s values and their individual and classroom-level trajectories of change aligned with the value structure proposed by Schwartz (1992). While self-transcendence values showed an increase, the conflicting values of self-enhancement showed a decrease over time. These results confirm previous research, which indicated that children’s values were similar to adults, with self-transcendence values the most important and self-enhancement the least important (Twito & Knafo-Noam, 2020). Further, while the importance of openness to change values increased over time, there was a decrease in the importance of the conflicting values of conservation. This is in accordance with previous research (e.g., Cieciuch et al., 2016; Daniel et al., 2023), which also found that in middle childhood, children increase their prioritization of independence, curiosity, and a readiness for change, over values that are associated with maintaining the status quo, preserving traditions, and conforming to societal expectations. This pattern can be associated with children’s cognitive growth and transition into adolescence, a phase distinguished by identity exploration (Harter, 1999).

One rationale to explain children’s value development within the school environment was that children’s values serve as a driving force behind their actions, i.e., they motivate their behavior (Schwartz, 1994). Accordingly, we analyzed children’s individual and classroom-level trajectories of change in behaviors. Contrary to our expectations, we did not find that children’s individual trajectories of change in behaviors followed the same structure as their values. Specifically, while the frequencies of supportive, disciplined and learning-oriented behaviors decreased within our study period, only achievement-oriented behavior increased in frequency. Our findings, based on teachers'ratings of student behavior, may indicate that the increase in children's achievement-oriented behavior might reflect the growing emphasis on academic performance in elementary schools, potentially encouraging even the youngest pupils to adopt a competitive mindset. Teachers may be more attuned to and likely to report achievement-oriented behaviors as academic expectations intensify. Conversely, the observed decrease in supportive, disciplined, and learning-oriented behaviors could indicate challenges in maintaining these behaviors as academic pressures increase. It is possible that teachers’ perceptions and ratings of these behaviors are influenced by the changing academic environment, potentially leading to a shift in focus towards achievement-oriented actions at the expense of other important behavioral aspects (see Neuenschwander & Makarova, 2024). Considering classroom-level trajectories of change in children’s behavior, we found that all four behaviors remained constant throughout the study. Furthermore, the duration of our study could have been insufficient to capture the structured change proposed by Schwartz’s value theory (1992). These factors could explain the unexpected patterns we observed in behavioral change structures compared to value trajectories.

### Time-lagged associations between children’s values and teacher-rated classroom behaviors

Our second objective was to examine time-lagged associations between children’s values and teacher-rated classroom behaviors over time. We confirmed our hypotheses about the temporal associations between behaviors and values seven times out of eight, examining how each domain at previous time points was statistically related to the trajectory in the other domain at subsequent time points, beyond what could be explained by their respective developmental trajectories. This analysis aligns with previous research on the dynamic interplay between values and behaviors over time (e.g., Aquilar et al., 2018; Vecchione et al., 2016a). We initially asked: How do values predict future behaviors (specifically behavior at the next time point) in addition to what is explained by their respective developmental trajectories, and conversely, how does behavior predict future values (specifically values at the next time point) in addition to what is explained by their respective developmental trajectories? Our findings clearly confirm the reciprocal nature of the complex associations in children’s value-behavior predictions over time. Children’s values significantly positively predicted the respective behavior one time point later, except for conservation values which did not significantly predict future disciplined behavior. Furthermore, the higher children’s supportive, achievement-, learning-oriented, and disciplined behavior was, the higher were their future values of self-transcendence, self-enhancement, openness to change, and conservation respectively. Nevertheless, our overall results affirm that the developmental trajectories of values and behaviors are interdependent throughout time. Previous research with older children found longitudinal links in both directions, but with a stronger influence from values to behavior (Benish-Weisman, 2015; Vecchione et al., 2016a). However, it was questioned whether this pattern might differ in young children due to developmental factors. Our results highlight the bidirectional time-lagged associations between children’s values and teacher-rated classroom behaviors in both directions, emphasizing the bidirectional predictive relationship between children’s and their classroom behaviors. The findings highlight the interconnected and dynamic nature of these factors, suggesting a reciprocal process of mutual influence in the school context.

A possible explanation for the direction from behaviors to time-lagged value trajectories might be the younger age of the sample, an age, in which the individual stability of children’s values is still in the process of improving as they grow older (Cieciuch et al., 2016). Further, middle childhood is an age, in which children start to strive toward continuity of their self (Klimstra, 2012), their personality is still being developed and accordingly different personality aspects, such as a parallel development of values and behavior may influence each other (see Vecchione et al., 2016a), which could also be a possible explanation for the complexity of value-behavior relations of children and its dynamic and changing nature over time (Jacobs et al., 2002). This finding supports the initial consideration that behaviors, being more concrete, might have a stronger influence on subsequent value trajectories at this early developmental stage. The dynamic interplay we observed suggests that contrary to findings in adolescence (e.g., Benish-Weisman, 2015), children’s abstract value concepts did not play a more significant role in predicting their concrete actions. These findings highlight the complexity of early personality development and underscore the importance of considering both values and behaviors in understanding children’s developmental trajectories.

On the other hand, the direction from values to time-lagged behavior trajectories might suggest that even young children’s abstract concepts can significantly guide their actions and potentially contribute to their future selves. This pattern implies a developmental process wherein early formed values play a crucial role in guiding behavior, which may then reinforce or slightly modify these values over time. Such a process could contribute to the formation of a more integrated sense of self as children develop. However, regardless of direction, the bidirectional nature of the relationship might be most important for educational practice and future research. It is important to note that the differences in our findings compared to previous studies may be attributed not only to the younger age of our sample but also to our unique analytical approach. Furthermore, the multilevel structure of our analysis accounts for the nested nature of our data (children within classrooms), providing more accurate estimates of the relationships between values and behaviors within the school context. This sophisticated approach is particularly well-suited for capturing the dynamic nature of value-behavior relationships in young children, where both constructs are still developing and potentially influencing each other in complex ways. The increased sensitivity of our analytical method in detecting bidirectional effects in younger children, combined with its ability to account for both individual trajectories and time-specific influences simultaneously, may explain why we found stronger bidirectional effects compared to studies with older children or those using different methodologies. These methodological considerations are crucial for interpreting our results and understanding how they contribute to the broader literature on value-behavior relationships across different developmental stages.

### Limitations and directions for future research

Future research should explore the factors that contribute to the formation of these influential early values. This could provide important insights for understanding and potentially guiding children’s developmental trajectories. Experimental designs could further investigate causality and practical applications (see Haney & Durlak, 1998). For instance, promoting prosocial values in classrooms might reduce disruptive behavior and enhance positive social outcomes (Abramson et al., 2018). Other possibilities to promote prosocial values are school intervention programs, which often aim to cultivate empathy, cooperation and a sense of responsibility in individuals, such as school programs that are focusing on values such as kindness or respect, social-emotional learning programs, which aim to enhance social and emotional skills in individuals and promote self-awareness, or anti-bullying programs, which aim to create a positive and inclusive social environment, discouraging aggressive behavior. By providing a theoretical foundation to understand and internalize prosocial behavior, values play a crucial role in these programs.

Contextual factors such as the school environment (e.g., Berson & Oreg, 2016) and peer interactions (e.g., Benish-Weisman et al., 2022; Benish-Weisman, 2024) likely play a significant role in maintaining individual differences over time. This understanding could inform proactive classroom management strategies. The challenge now lies in translating this empirical knowledge into practical applications for educators. Regardless of direction, the bidirectional nature of the relationship might be most important for educational practice. These findings have important implications for early intervention strategies and educational practices, suggesting that targeting both values and behaviors could potentially influence children’s developmental trajectories. Future research could delve deeper into the mechanisms underlying reciprocal value-behavior trajectories over time. It would be valuable to investigate whether this pattern holds across different types of values and behaviors or varies at different stages of early childhood development. Importantly, we confirmed the bidirectional nature of the associations between values and behaviors, indicating that their developmental trajectories are interdependent throughout time, suggesting that values and behaviors develop in tandem, each influencing the other’s development. Based on our findings, we suggest that teachers, as key figures in young children’s development, should be aware of the developmental interplay between children’s values and behaviors in the classroom over time. Additionally, this knowledge should be applied in proactive classroom management, aligning with findings that teachers prioritize conformity as a goal (see Scholz-Kuhn et al., 2021).

While this study provides valuable insights, it is important to acknowledge certain limitations that should be addressed in future research. First, we examined changes in values, behavior and their relations over relatively short time spans (three to four months), which can be justified by previous findings with children (e.g., Vecchione et al., 2016a). Nevertheless, the timing of data collection needs to be further investigated, i.e., examining the effects of values and behaviors on one another in the classroom over longer periods of time, especially as previous value change research in adults has found some delayed effects (Daniel et al., 2022). Second, the use of scales for measuring behavior (i.e., teacher-rated behavior) is a common practice and their validity has been shown (e.g., Benish-Weisman et al., 2022; Berson & Oreg, 2016). Moreover, teachers did not report a particular explicit behavior; instead, they likely based their rating on a compilation of behaviors. In this regard, the measurement resembles an assessment of a trait, and this could explain the robust consistency of the behavioral measure over time. In fact, this is what makes this measure of behaviors so suitable to correlate with values, which are also trait and not state-related variables. Since we used children’s self-reports (for the values) and teacher-rating (for the behaviors) common method variance (see Podzakoff et al., 2003) was avoided. However, it is crucial to consider the strengths and limitations of each method, and future research would benefit by triangulating information from multiple sources, such as observed behavior, for a more accurate assessment and to provide the full range of relationships and effects.

## Conclusion

This research offers insights into the bidirectional longitudinal relationships between values and behaviors of young children at the start of their schooling. By following children through their first two years of elementary school, we analyzed a crucial developmental period marked by significant social and cognitive changes. Our findings confirm that children’s values are aligned with their behavior as proposed by Schwartz (1992, 1994), showing increases in self-transcendence and openness to change, and decreases in self-enhancement and conservation. Additionally, we identified bidirectional time-lagged associations, indicating that values and behaviors are related to each other over time. In summary, our study highlights the dynamic and interdependent nature of values and behaviors in young children’s development, emphasizing the need to consider both aspects in educational contexts. This understanding provides valuable implications for both educational practice and future research.

## Supplementary Information

Below is the link to the electronic supplementary material.

Supplementary file1 (DOCX 265 KB)

## Data Availability

The datasets analyzed during the current study are available from the corresponding author on reasonable request.
