# Supplementary material for: Children’s personal values and their behavior in the classroom in the early elementary school years: mapping longitudinal trajectories
Source: Eur J Psychol Educ. 2025 Jun 23;40(3):75. doi: 10.1007/s10212-025-00966-2 (PMC12185584; doi:10.1007/s10212-025-00966-2)
Supplement: Supplementary file 1 — Supplementary file1 (DOCX 265 KB) [file 10212_2025_966_MOESM1_ESM.docx]

**SUPPLEMENTARY MATERIAL**

**Code S1**

**Commented R code (November 21, 2024):**

#1. Set working directory and load data - according to instructions

setwd("C:/Users/ricarda.scholz/OneDrive - FHNW/Documents/Analyses")

getwd() #check that the folder is set right

# 2. Installing "pacman"

install.packages("pacman")

# Now further packages can be installed and loaded

pacman::p_load(lavaan, lme4,tidyr, nlme, tidyverse, lmerTest, ggplot2, ggthemes, foreign, plotrix, svd, psych, MASS, car, dplyr, apaTables, haven)

## 3. Data

df <- read_sav("data_final.sav")

## 3.1. Preparing subdata set

df_red <- df %>%

dplyr::select(id, SeTr_t1, SeTr_t2, SeTr_t3, SeTr_t4,

SeEn_t1, SeEn_t2, SeEn_t3, SeEn_t4,

Con_t1, Con_t2, Con_t3, Con_t4,

OtC_t1, OtC_t2, OtC_t3, OtC_t4,

cSupportive_t1,cSupportive_t2, cSupportive_t3, cSupportive_t4,

cDisciplined_t1, cDisciplined_t2, cDisciplined_t3, cDisciplined_t4,

cAchievement_t1, cAchievement_t2, cAchievement_t3, cAchievement_t4,

cLearning_oriented_t1, cLearning_oriented_t2, cLearning_oriented_t3, cLearning_oriented_t4,

gender,

class)

# 3.2 Making factors out of id and class (cluster variables)

id<-df_red$id

class<-df_red$class

# 3.3 Transforming wide to long

df_long<-reshape(df_red, direction='long',

varying=list(

SeTr=c("SeTr_t1","SeTr_t2","SeTr_t3",

"SeTr_t4"),

SeEn=c("SeEn_t1","SeEn_t2","SeEn_t3",

"SeEn_t4"),

OtC=c("OtC_t1","OtC_t2","OtC_t3",

"OtC_t4"),

Con=c("Con_t1","Con_t2","Con_t3",

"Con_t4"),

cDisc=c("cDisciplined_t1","cDisciplined_t2",

"cDisciplined_t3","cDisciplined_t4"),

cSupp=c("cSupportive_t1","cSupportive_t2",

"cSupportive_t3","cSupportive_t4"),

cLear=c("cLearning_oriented_t1","cLearning_oriented_t2",

"cLearning_oriented_t3","cLearning_oriented_t4"),

cAchi=c("cAchievement_t1","cAchievement_t2","cAchievement_t3",

"cAchievement_t4")),

timevar='time',

times=c("1", '2',"3","4"),

v.names=c('SeTr', 'SeEn',"OtC","Con","cDisc","cSupp",

"cLearn","cAchi"),

idvar=c('id', "class"))

# 3.4 First we need to recode as numeric

df_long$SeTr<-as.numeric(df_long$SeTr)

df_long$SeEn<-as.numeric(df_long$SeEn)

df_long$OtC<-as.numeric(df_long$OtC)

df_long$Con<-as.numeric(df_long$Con)

df_long$cDisc<-as.numeric(df_long$cDisc)

df_long$cSupp<-as.numeric(df_long$cSupp)

df_long$cLearn<-as.numeric(df_long$cLearn)

df_long$cAchi<-as.numeric(df_long$cAchi)

df_long$time<-as.numeric(df_long$time)

df_long$time2<-df_long$time*df_long$time

# 3.5 Center variables SeTr, SeEn, OtC, and Con around 0, replacing the original variables

df_long_sortiert <- df_long_sortiert %>%

mutate(across(c(SeTr, SeEn, OtC, Con), ~ . - 3))

# List of your variables

variables <- c("SeTr", "SeEn", "OtC", "Con", "cDisc", "cSupp", "cLearn", "cAchi")

# 3.6 Calculate class means for each time point

df_long_sortiert <- df_long_sortiert %>%

group_by(class, time) %>%

mutate(across(all_of(variables),

~ mean(., na.rm = TRUE),

.names = "class_mean_{.col}")) %>%

ungroup()

# 3.7 Calculate person-mean centered values

df_long_sortiert <- df_long_sortiert %>%

group_by(class, id) %>%

mutate(across(all_of(variables),

list(person_mean = ~ mean(., na.rm = TRUE),

person_centered = ~ . - mean(., na.rm = TRUE)))) %>%

ungroup()

# 3.8 Calculate the deviation from class mean at each time point

df_long_sortiert <- df_long_sortiert %>%

mutate(across(all_of(variables),

~ . - get(paste0("class_mean_", cur_column())),

.names = "deviation_from_class_mean_{.col}"))

##4. preparing data for future analyses

#4.1 Sorting the data: class - id - time

df_long_sortiert <- df_long[order(df_long$class, df_long$id, df_long$time),]

# 4.2 Because every person has 4 time points, all we need to do is delete the first time point and add an NA to the end.

SeTrstorage<-c(df_long_sortiert$SeTr[2:nrow(df_long_sortiert)],NA)

SeEnstorage<-c(df_long_sortiert$SeEn[2:nrow(df_long_sortiert)],NA)

OtCstorage<-c(df_long_sortiert$OtC[2:nrow(df_long_sortiert)],NA)

Constorage<-c(df_long_sortiert$Con[2:nrow(df_long_sortiert)],NA)

Disciplinedstorage<-c(df_long_sortiert$cDisc[2:nrow(df_long_sortiert)],NA)

Supportivestorage<-c(df_long_sortiert$cSupp[2:nrow(df_long_sortiert)],NA)

Achievementstorage<-c(df_long_sortiert$cAchi[2:nrow(df_long_sortiert)],NA)

Learningstorage<-c(df_long_sortiert$cLearn[2:nrow(df_long_sortiert)],NA)

# 4.3 then merge

df_long_sortiert$futureSeTr<-SeTrstorage

df_long_sortiert$futureSeEn<-SeEnstorage

df_long_sortiert$futureOtC<-OtCstorage

df_long_sortiert$futureCon<-Constorage

df_long_sortiert$futureDisciplined<-Disciplinedstorage

df_long_sortiert$futureSupportive<-Supportivestorage

df_long_sortiert$futureAchievement<-Achievementstorage

df_long_sortiert$futureLearning<-Learningstorage

# 4.4 Then replace every 4th time point with NA, as we do not have a future value for that time point.

df_long_sortiert$futureSeTr[df_long_sortiert$time==4]<-NA

df_long_sortiert$futureSeEn[df_long_sortiert$time==4]<-NA

df_long_sortiert$futureOtC[df_long_sortiert$time==4]<-NA

df_long_sortiert$futureCon[df_long_sortiert$time==4]<-NA

df_long_sortiert$futureDisciplined[df_long_sortiert$time==4]<-NA

df_long_sortiert$futureSupportive[df_long_sortiert$time==4]<-NA

df_long_sortiert$futureAchievement[df_long_sortiert$time==4]<-NA

df_long_sortiert$futureLearning[df_long_sortiert$time==4]<-NA

# 5. Centering of time and time-squared

df_long_mean <- df_long_mean %>%

mutate(

time_c = time - 1, # or time - mean(time) for mean centering

time2_c = (time_c)^2)

# 6. ICCs

misty::multilevel.descript(df_long_sortiert[, c("SeTr")], cluster = df_long_sortiert$class)

misty::multilevel.descript(df_long_sortiert[, c("SeEn")], cluster = df_long_sortiert$class)

misty::multilevel.descript(df_long_sortiert[, c("Con")], cluster = df_long_sortiert$class)

misty::multilevel.descript(df_long_sortiert[, c("OtC")], cluster = df_long_sortiert$class)

misty::multilevel.descript(df_long_sortiert[, c("cSupp")], cluster = df_long_sortiert$class)

misty::multilevel.descript(df_long_sortiert[, c("cAchi")], cluster = df_long_sortiert$class)

misty::multilevel.descript(df_long_sortiert[, c("cDisc")], cluster = df_long_sortiert$class)

misty::multilevel.descript(df_long_sortiert[, c("cLearn")], cluster = df_long_sortiert$class)

misty::multilevel.descript(df_long_sortiert[, c("class_mean_SeTr")], cluster = df_long_sortiert$class)

misty::multilevel.descript(df_long_sortiert[, c("class_mean_SeEn")], cluster = df_long_sortiert$class)

misty::multilevel.descript(df_long_sortiert[, c("class_mean_Con")], cluster = df_long_sortiert$class)

misty::multilevel.descript(df_long_sortiert[, c("class_mean_OtC")], cluster = df_long_sortiert$class)

misty::multilevel.descript(df_long_sortiert[, c("class_mean_cSupp")], cluster = df_long_sortiert$class)

misty::multilevel.descript(df_long_sortiert[, c("class_mean_cAchi")], cluster = df_long_sortiert$class)

misty::multilevel.descript(df_long_sortiert[, c("class_mean_cDisc")], cluster = df_long_sortiert$class)

misty::multilevel.descript(df_long_sortiert[, c("class_mean_cLearn")], cluster = df_long_sortiert$class)

# 7. Optimizer to support model convergence and precision, contains a list of control parameters for the optimization algorithm

list<-lmeControl(maxIter=1000,msMaxIter = 1000, niterEM = 1000,msMaxEval = 1000,opt = "optim")

# 5. Employing multilevel growth curve analysis using the R package nlme

# **Research objective 1: *Identifying individual and classroom-level trajectories of change in values and behaviors in middle childhood within the school context by employing multilevel growth curve analysis***

# Let's check which growth model provides better fit for the standard progression of stress - linear or quadratic

# Starting with values as outcome and corresponding behaviors as predictors

**# Self-transcendence (example) – individual trajectory of change**

# No Growth Baseline

Mod100<-lme(data=df_long_mean,SeTr~1,random =~ 1|class/id,na.action = na.omit,method = "ML")

summary(Mod100)

#linear

Mod101<-lme(data=df_long_mean,SeTr~time,random =~ 1+time_c|class/id,na.action = na.omit,method = "ML",control = list)

summary(Mod101)

#check

anova(Mod100,Mod101) # significant difference - linear wins.

#Second, let's check if a linear or quadratic growth model is more fitting

#quadratic

Mod102<-lme(data=df_long_mean,SeTr~time_c+time2_c,random =~ 1+time_c+time2_c|class/id,na.action = na.omit,method = "ML",control = list)

summary(Mod102)

#check

anova(Mod101,Mod102) # significant difference - quadratic wins.

**# Self-transcendence (example) – classroom-level trajectory of change**

# No Growth Baseline

Mod100<-lme(data=df_long_mean, class_mean_SeTr~1,random =~ 1|class/id,na.action = na.omit,method = "ML")

summary(Mod100)

#linear

Mod101<-lme(data=df_long_mean, class_mean_SeTr~time,random =~ 1+time_c|class/id,na.action = na.omit,method = "ML",control = list)

summary(Mod101)

#check

anova(Mod100,Mod101) # significant difference - linear wins.

#Second, let's check if a linear or quadratic growth model is more fitting

#quadratic

Mod102<-lme(data=df_long_mean, class_mean_SeTr~time_c+time2_c,random =~ 1+time_c+time2_c|class/id,na.action = na.omit,method = "ML",control = list)

summary(Mod102)

#check

anova(Mod101,Mod102) # significant difference - quadratic wins.

**#Research objective 2: *Analyzing how behaviors from previous time points contribute to the trajectory of values at later time points, above and beyond what could be explained by the values’ own trajectories, and conversely, how values from previous time points contribute to the trajectories of behaviors at later time points, beyond what could be explained by the behaviors’ own trajectories* (the “future” implies the time-lagged outcome, i.e. we symbolize that we predict subsequent trajectories in values/behaviors)**

# Let's check which growth model provides better fit for the standard progression of stress - linear or quadratic to reach objective 2 (similar as above, but with time-lagged outcome variables, i.e., T2 – T4)

#No growth baseline

Mod200<-lme(data=df_long_mean,futureSeTr~1,random =~ 1|class/id,na.action = na.omit,method = "ML")

summary(Mod200)

#linear

Mod201<-lme(data=df_long_mean,futureSeTr~time_c,random =~ 1+time_c|class/id,na.action = na.omit,method = "ML")

summary(Mod201)

#check

anova(Mod200,Mod201) # significant difference - linear wins.

#Second, let's check if a linear or quadratic growth model is more fitting

#quadratic

Mod202<-lme(data=df_long_mean,futureSeTr~time_c+time2_c,random =~ 1+time_c+time2_c|class/id,na.action = na.omit,method = "ML",control = list)

summary(Mod202)

#check

anova(Mod201,Mod202) # significant difference - quadratic wins.p(<.0001)

#Adding the predictors (corresponding behavior raw (between-person effects) and centered (within-person effects) to the model

#Model supportive behavior as predictor

Mod300<-lme(data=df_long_mean,futureSeTr~time_c+time2_c+

cSupp,

random =~ 1+time_c+time2_c|class/id,na.action = na.omit,method = "ML",control = list)

summary(Mod300)

*Mod300 explained:*

- *Mod300 <-: This assigns the model output to an object named "Mod102".*
- *lme(): This function fits a linear mixed-effects model.*
- *data = df_long_mean: Specifies the dataset being used, which is in long format.*
- *futureSeTr ~ time_c + time2_c* *+ cSupp: This is the fixed effects formula.*
  - *futureSeTr is the dependent variable. (future represents the later, time-lagged SeTr values, predicting subsequent values)*
  - *time_c and time2_c are predictors, likely representing linear and quadratic time effects.*
  - *Supp,is a predictor, representing the time-varying supportive behavior (specific to each time point)*
- *random = ~ 1 + time_c + time2_c | class/id: This specifies the random effect’s structure.*
  - *It allows for random intercepts and random slopes for both linear and quadratic time.*
  - *The class/id notation indicates nested random effects: individuals (id) nested within classes.*
- *na.action = na.omit: This tells R to omit any rows with missing data.*
- *method = "ML": Specifies Maximum Likelihood estimation, which is appropriate for comparing models with different fixed effects.*
- *control = list: Adding the optimizer (see above) for the optimization algorithm*

Table S1

*Intercorrelations across time between self-enhancement, self-transcendence, achievement-oriented, supportive behaviors*

| Variable | 2 | 3 | 4 | 5 | 6 | 7 | 8 | 9 | 10 | 11 | 12 | 13 | 14 | 15 | 16 |
| --- | --- | --- | --- | --- | --- | --- | --- | --- | --- | --- | --- | --- | --- | --- | --- |
| 1. SeEn T1 | .53*** | .41*** | .46*** | -.52*** | -.29*** | -.31*** | -.27*** | .14*** | .18*** | .15*** | .12** | -.05 | -.11** | -.10** | -.02 |
| 2 SeEn T2 |  | .62*** | .58*** | -.37*** | -.57*** | -.46*** | -.37*** | .12*** | .17*** | .15*** | .16*** | -.02 | -.12*** | -.07* | -.06 |
| 3 SeEn T3 |  |  | .65*** | -.32*** | -.39*** | -.52*** | -.38*** | .10** | .17*** | .16*** | .17*** | -.03 | -.14** | -.10** | -.10** |
| 4SeEn T4 |  |  |  | -.35*** | -.39*** | -.42*** | -.53*** | .15*** | .17*** | .17*** | .17*** | -.09* | -.13*** | -.09** | -.08** |
| 5 SeTr T1 |  |  |  |  | .40*** | .44*** | .38*** | -.11** | -.19*** | -.12** | -.17*** | .08* | .11** | .07 | .06 |
| 6 SeTr T2 |  |  |  |  |  | .57*** | .47*** | -.13*** | -.15*** | -.14*** | -.15*** | 0.06 | .12*** | .07* | .07* |
| 7 SeTr T3 |  |  |  |  |  |  | .60*** | -.13*** | -.16*** | -.15*** | -.18*** | .10* | .11** | .09** | .10** |
| 8 SeTr T4 |  |  |  |  |  |  |  | -.16*** | -.13*** | -.16*** | -.18*** | .12** | .09** | .08* | .08* |
| 9 Achi T1 |  |  |  |  |  |  |  |  | .63*** | .58*** | .54*** | -.73*** | -.50*** | -.48*** | -.40*** |
| 10 Achi T2 |  |  |  |  |  |  |  |  |  | .63*** | .64*** | -.52*** | -.74*** | -.50*** | -.51*** |
| 11 Achi T3 |  |  |  |  |  |  |  |  |  |  | .72*** | -.48*** | -.53*** | -.70*** | -.53*** |
| 12 Achi T4 |  |  |  |  |  |  |  |  |  |  |  | -.46*** | -.56*** | -.55*** | -.68*** |
| 13 Supp T1 |  |  |  |  |  |  |  |  |  |  |  |  | .61*** | .60*** | .52*** |
| 14 Supp T2 |  |  |  |  |  |  |  |  |  |  |  |  |  | .64*** | .64*** |
| 15 Supp T3 |  |  |  |  |  |  |  |  |  |  |  |  |  |  | .63*** |
| 16 Supp T4 |  |  |  |  |  |  |  |  |  |  |  |  |  |  |  |

*Note*. * *p* < .05. ** *p* < .01. *** *p* < .001.

Abbreviations: SeTr = self-transcendence, SeEn = self-enhancement, Supp = supportive behavior (centered), Achi = achievement-oriented behavior (centered); *N*s are ranging from 834 to 1184.

Table S2

Intercorrelations across time between conservation, openness to change, disciplined, learning-oriented behaviors

| Variable | 2 | 3 | 4 | 5 | 6 | 7 | 8 | 9 | 10 | 11 | 12 | 13 | 14 | 15 | 16 |
| --- | --- | --- | --- | --- | --- | --- | --- | --- | --- | --- | --- | --- | --- | --- | --- |
| 1 Con T1 | .33*** | .21*** | .32*** | -.50*** | -.17*** | -.16*** | -.21*** | .16*** | .06 | .13*** | .12** | -.08* | -.05 | -.11** | -.08 |
| 2 Con T2 |  | .49*** | .49*** | -.27*** | -.60*** | -.36*** | -.36*** | .07* | .07* | .10*** | .11*** | -.05 | -.06 | -.09** | -.10** |
| 3 Con T3 |  |  | .58*** | -.24*** | -.35*** | -.61*** | -.40*** | .07 | .11*** | .12*** | .12*** | -.10** | -.15*** | -.18*** | -.15*** |
| 4 Con T4 |  |  |  | -.29*** | -.42*** | -.45*** | -.66*** | .11** | .11*** | .12*** | .15*** | -.13*** | -.16*** | -.15*** | -.18*** |
| 5 OtC T1 |  |  |  |  | .42*** | .39*** | .39*** | -.09*** | -.04 | -.12** | -.13*3* | .12*** | .05 | .12** | .11** |
| 6 OtC T2 |  |  |  |  |  | .54*** | .55*** | -.09* | -.09** | -.14*** | -.13*** | .14*** | .12*** | .16*** | .17*** |
| 7 OtC T3 |  |  |  |  |  |  | .62*** | -.09* | -.10*** | -.09** | -.11*** | .18*** | .14*** | .16*** | .14*** |
| 8 OtC T4 |  |  |  |  |  |  |  | -.11** | -.10*** | -.14*** | -.15*** | .16*** | .15*** | .17*** | .18*** |
| 9 Disc T1 |  |  |  |  |  |  |  |  | .64*** | .61*** | .63*** | -.49*** | -.37*** | -.35*** | -.42*** |
| 10 Disc T2 |  |  |  |  |  |  |  |  |  | .63*** | .67*** | -.47*** | -.53*** | -.36*** | -.50*** |
| 11 Disc T3 |  |  |  |  |  |  |  |  |  |  | .69*** | -.40*** | -.42*** | -.43*** | -.46*** |
| 12 Disc T4 |  |  |  |  |  |  |  |  |  |  |  | -.42*** | -.44*** | -.41*** | -.57*** |
| 13 Learn T1 |  |  |  |  |  |  |  |  |  |  |  |  | .61*** | .57*** | .54*** |
| 14 Learn T2 |  |  |  |  |  |  |  |  |  |  |  |  |  | .58*** | .61*** |
| 15Learn T3 |  |  |  |  |  |  |  |  |  |  |  |  |  |  | .61*** |
| 16 Learn T4 |  |  |  |  |  |  |  |  |  |  |  |  |  |  |  |

*Note*. * *p* < .05. ** *p* < .01. *** *p* < .001.

Abbreviations: Con = conservation, OtC = openness to change, Disc = disciplined behavior (centered), Learn = learning-oriented behavior (centered); *N*s are ranging from 834 to 1184.


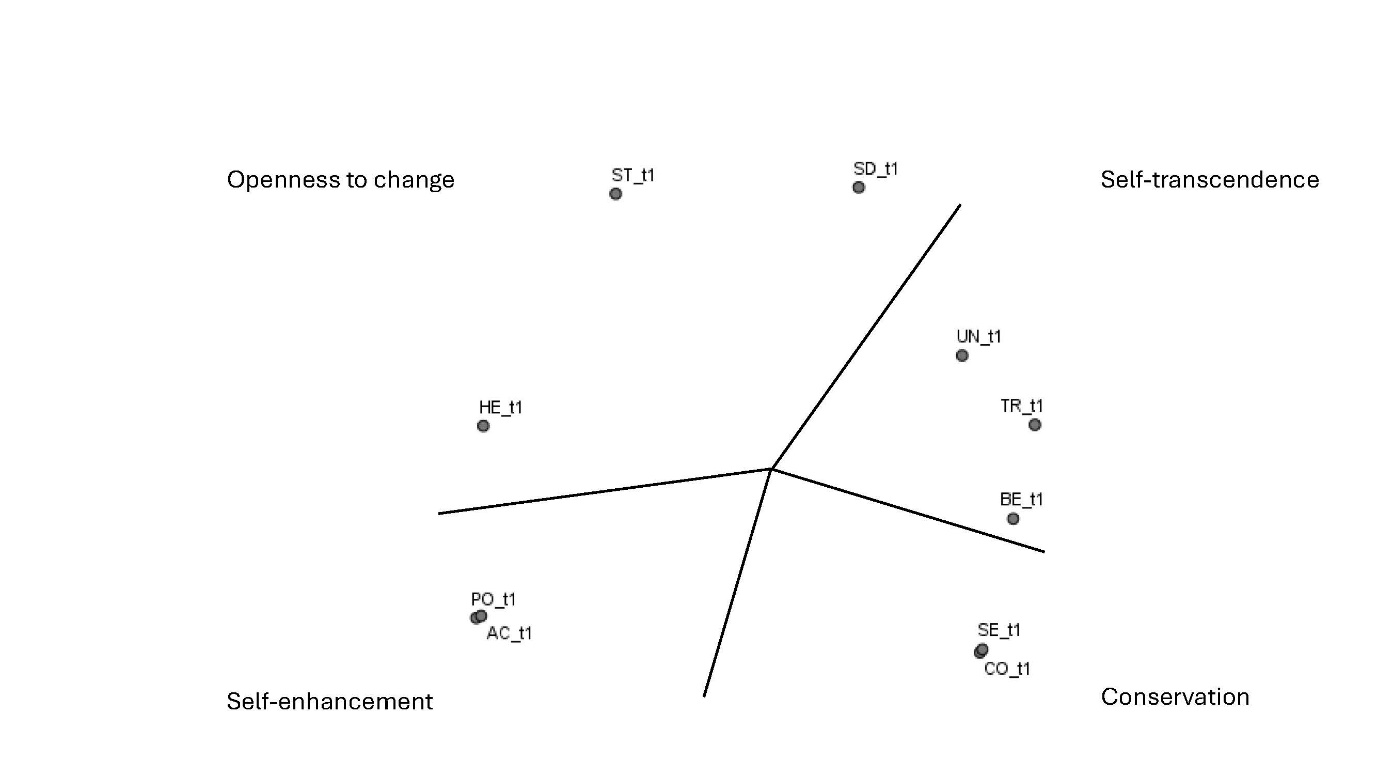
Figure S1

*Multidimensional scaling of children’s values operationalized through 20 items from PBVS-C (Author et al., 2010), subsumed to the four higher-order values. Each point represents two items of the same basic value (Stress 1 =.09). UN = Universalism, BE = Benevolence, TR = Tradition, CO = Conformity, SE = Security, AC = Achievement, PO = Power, HE = Hedonism, ST = Stimulation, SD = Self-direction.*


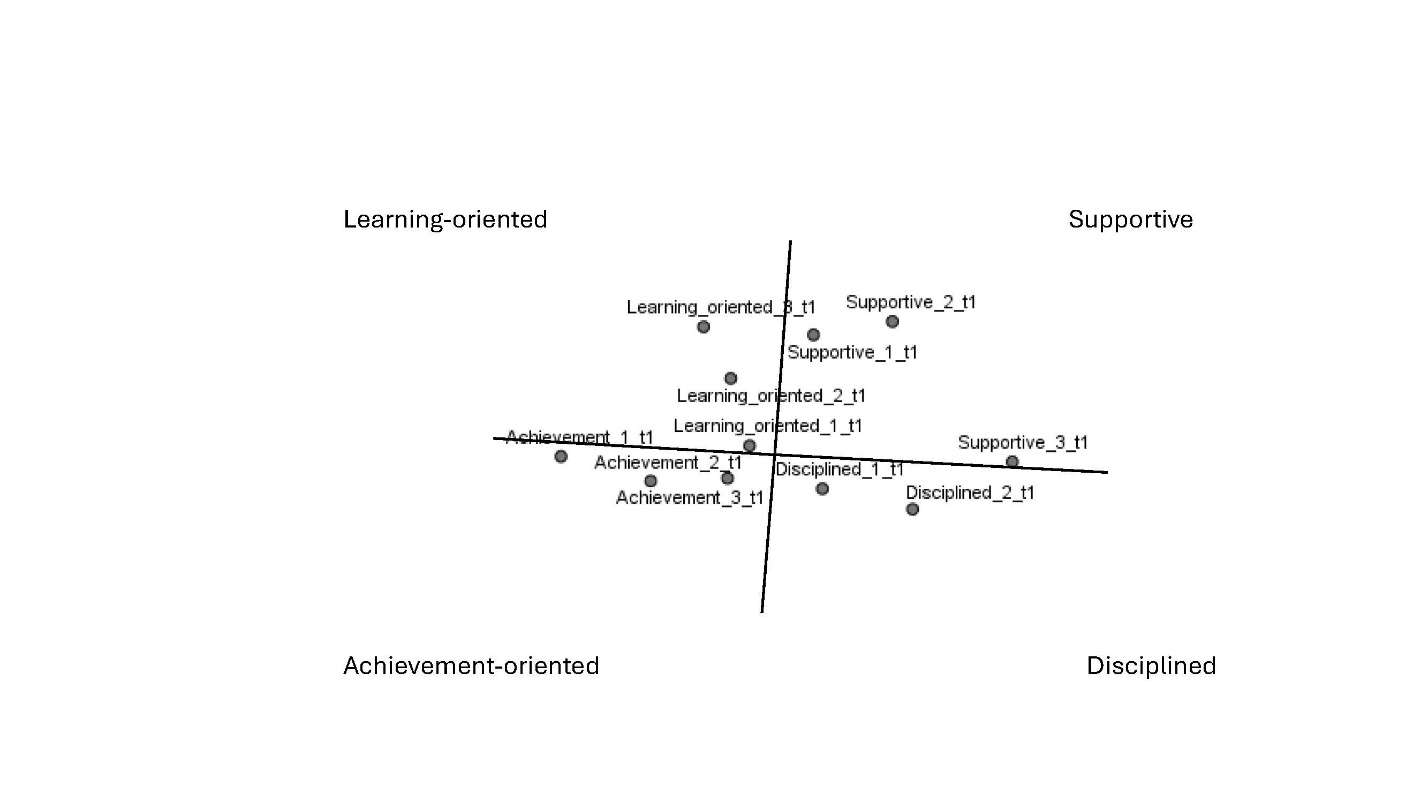


Figure S2

*Multidimensional scaling of children’s behavior operationalized through 11 items of the behavior scale (Berson & Oreg, 2016) (three items for learning-oriented, supportive and achievement-oriented, two items for disciplined). (Stress 1 =.05)*

Openness to change Self-transcendence


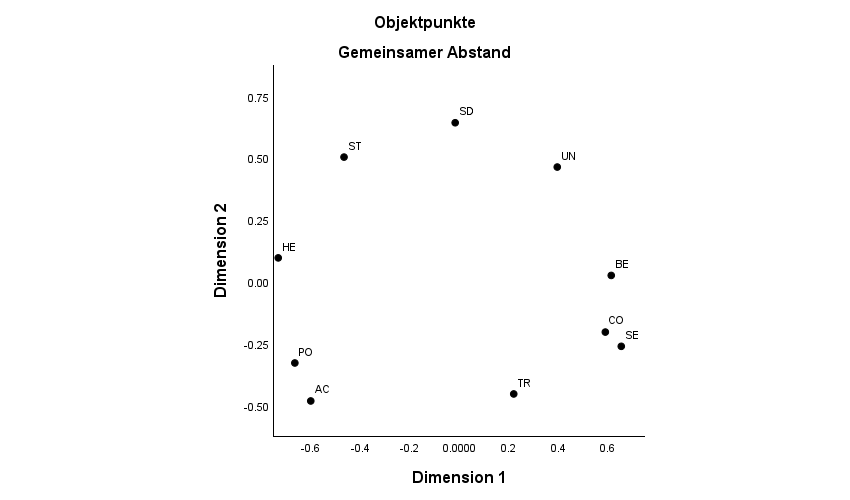


Self-enhancement Conservation

Figure S3

*MDS Values at last point of measurement (Stress 1 =.09). UN = Universalism, BE = Benevolence, TR = Tradition, CO = Conformity, SE = Security, AC = Achievement, HE = Hedonism, ST = Stimulation, SD = Self-direction.*

Learning-oriented Supportive


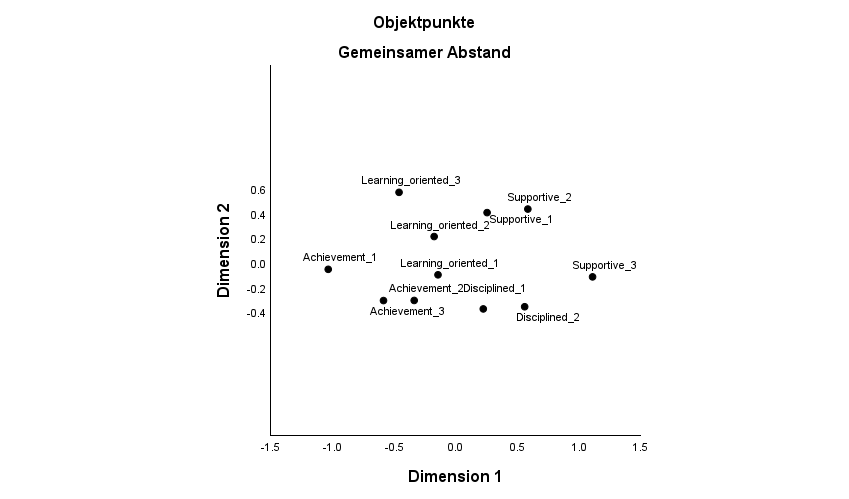


Achievement-oriented Disciplined

Figure S4

*MDS of children’s behavior at last point of measurement (Stress 1 =.04)*
